# Evaluating the impact of coenzyme Q10 and high-intensity interval training on lactate threshold and Plasma blood gases in rats: a randomized controlled trial

**DOI:** 10.1007/s00421-025-05756-8

**Published:** 2025-03-18

**Authors:** Yavuz Yasul, Büşra Yılmaz, Ömer Şenel, Dursun Kurt, Taner Akbulut, Ayşen Çalıkuşu, Elvan Anadol, Canan Yılmaz

**Affiliations:** 1 Bafra Vocational School, Ondokuz Mayıs University, 55400 Bafra, Samsun Türkiye; 2 Faculty of Sport Sciences, Gazi University, Ankara, Türkiye; 3 Faculty of Sport Sciences, Fırat University, Elazığ, Türkiye; 4 Department of Anatomy, Ege University, Izmir, Türkiye; 5 Laboratory Animals Breeding and Experimental Researches Center, Gazi University, Ankara, Türkiye; 6 Faculty of Medicine, Department of Medical Biochemistry, Gazi University, Ankara, Türkiye

**Keywords:** Blood gases, Coenzyme Q_10_, HIIT, Lactate threshold, Oxygenation, Recovery

## Abstract

**Purpose:**

Coenzyme Q_10_ (Q_10_) is a mitochondrial coenzyme that facilitates ATP production via oxidative phosphorylation. This study hypothesized that Q_10_ enhances mitochondrial efficiency and lactate threshold, while high-intensity interval training (HIIT) promotes metabolic adaptations, improving glucose utilization and buffering capacity for faster recovery after high-intensity exercise.

**Methods:**

A randomized controlled trial was conducted using 24 male Sprague–Dawley rats (250.4 ± 6.1 g, 8 weeks old). The rats were allocated into four groups: control (C), coenzyme Q_10_ (CoQ_10_), HIIT, and HIIT + Q_10_. The Q_10_ administration involved a dosage of 10 mg/kg/day, given 30 min prior to the HIIT protocol. Lactate threshold, blood gas parameters, oximetry values, metabolite levels, and electrolyte status were analyzed utilizing the Radiometer 900 device. The blood samples were collected at the fifth and tenth minutes following the HIIT training trials.

**Results:**

The HIIT + Q_10_ group exhibited a significant reduction in lactate threshold (*p* < 0.01), maintaining values below average. Significant improvements in blood gas parameters, including pH, pO2, and pCO2, were observed in this group. Enhanced oxygen transport capacity was indicated by improved oximetry parameters (Hb, HCT, sO_2_) and reduced COHb levels. Additionally, positive changes in HCO_3_- and base values indicated reduced metabolic stress. Q_10_ supplementation also stabilized electrolytes, particularly K^+^ and Na^+^.

**Conclusion:**

The Q_10_ supplementation supported metabolic balance, improved oxygen transport, and stabilized acid–base levels during HIIT. It reduced lactate accumulation, enhanced glucose availability, and alleviated metabolic stress, thereby improving recovery efficiency and physiological adaptation.

**Supplementary Information:**

The online version contains supplementary material available at 10.1007/s00421-025-05756-8.

## Introduction

The greatest challenge for coaches and athletes is to identify the optimal combination of exercises during training sessions. Considering its positive effects, high-intensity interval training (HIIT) has recently emerged as one of the popular alternatives. Interval training is defined as the repetition of relatively intense efforts interspersed with periods of lighter activity or rest. This method is generally applied in the context of athletic performance and forms the foundation of training programs for elite athletes (Fox et al. 1973; Altinel et al. 2024).

The fundamental principle of HIIT is to accumulate a greater workload at a higher intensity than what can be achieved through continuous exercise at a steady intensity. The workload capacity developed through HIIT plays a critical role in achieving success in sports and activities such as middle and long-distance running, cycling, swimming, rowing, and cross-country skiing (Hellard et al. 2019; Leo et al. 2020; Torvik et al. 2021; Casado et al. 2022). This perspective is based on the assumption that HIIT strengthens physiological responses, enhances the ability to sustain higher workloads, and increases resistance to fatigue during competition (Laursen and Jenkins 2002). In this context, HIIT serves as a powerful stimulus that triggers physiological remodeling, enhances exercise performance, and improves oxidative capacity (Gibala and Jones 2013). In contrast, HIIT induces both mechanical and metabolic stress during the session (Leite et al. 2023). Unlike aerobic exercise, HIIT places a significantly greater demand on both aerobic and anaerobic energy systems. The repeated high-intensity efforts require rapid ATP resynthesis, leading to increased reliance on anaerobic glycolysis, and heightened metabolic acidosis due to accelerated lactate accumulation. Unlike continuous moderate-intensity exercise, where energy production remains stable, HIIT induces repeated metabolic fluctuations that challenge energy homeostasis. These extreme demands may necessitate additional physiological support to optimize recovery, buffer acidosis, and sustain performance across repeated high-intensity bouts (Maclnnis and Gibala 2017). The new metabolic load resulting from HIIT is monitored through indicators such as blood lactate threshold (Lac) levels and blood gas exchange parameters, which help determine the limits of exertion. Furthermore, the accumulation of Lac above its baseline level and the limitations in oxygen utilization define the boundaries of power and speed performance (Smith and Jones 2001). Therefore, plasma Lac levels and blood gas exchange markers reflect the highest sustainable intensity, where a significant increase in non-oxidative metabolism associated with exercise intolerance is observed (Poole et al. 2016).

Athletes frequently use dietary supplements to achieve high levels of athletic performance and to enhance sustainability by reducing metabolic intolerance (Mason et al 2020). Despite potential side effects, sodium bicarbonate, sodium citrate, and beta-alanine-based supplements are commonly preferred by athletes (Gencoglu et al. 2022). Sodium bicarbonate functions as an extracellular buffer, neutralizing hydrogen ions to delay fatigue, while beta-alanine enhances intracellular buffering by increasing muscle carnosine levels. However, both agents have limitations, including gastrointestinal discomfort and dosage sensitivity. Coenzyme Q_10_ (Q_10_), along with its various effects, functions as a coenzyme for mitochondrial enzymes in the electron transport system, playing a crucial role in energy production within the mitochondrial respiratory chain and facilitating ATP synthesis via oxidative phosphorylation. Additionally, Q_10_ contributes to lactate metabolism by enhancing the efficiency of the pyruvate dehydrogenase complex, promoting aerobic metabolism over anaerobic glycolysis. Furthermore, its antioxidant properties help mitigate oxidative stress, which can otherwise impair mitochondrial function during high-intensity exercise (Goncalves et al. 2015; Mason et al. 2020). Unlike traditional buffering agents, Q_10_ not only aids in pH regulation but also supports mitochondrial efficiency and energy availability, making it a multifunctional alternative with added metabolic benefits. This evidence strengthens the notion that Q_10_ could serve as a novel buffering alternative due to its bioenergetic role in muscle contraction, muscle-protective effects, and involvement in inflammatory processes (Drobnic et al. 2022). The studies focusing on this topic have reported that Q_10_ supplementation improved energy production and athletic performance while reducing lactate accumulation levels in male middle-distance runners who received 5 mg/kg for 14 days prior to competition (Armanfar et al. 2015), cyclists who were supplemented with 20 mg/kg for 12 days (Broome et al. 2021), and runners who consumed 60 mg/kg for 10 days (Ovchinnikov et al. 2022).

Research has generally focused on pre-exercise conditions, moderate-intensity exercise distributions, and short-term persistent responses. However, the effects of Q_10_ supplementation on lactate accumulation during high-intensity exercise conditions and HIIT have yet to be fully elucidated (Yasul et al. 2024). Additionally, the effects of Q_10_ supplementation on lactic acid accumulation and other plasma blood gases during HIIT have not been sufficiently clarified. Based on these considerations, we hypothesize that CoQ_10_ supplementation will facilitate a faster metabolic recovery post HIIT by lowering lactate levels, optimizing blood gas parameters and enhancing mitochondrial efficiency. This study aims to evaluate these effects by analyzing post-exercise recovery dynamics in rats undergoing HIIT with and without CoQ_10_ supplementation.

## Methods

### Experimental design

**Fig. 1:**
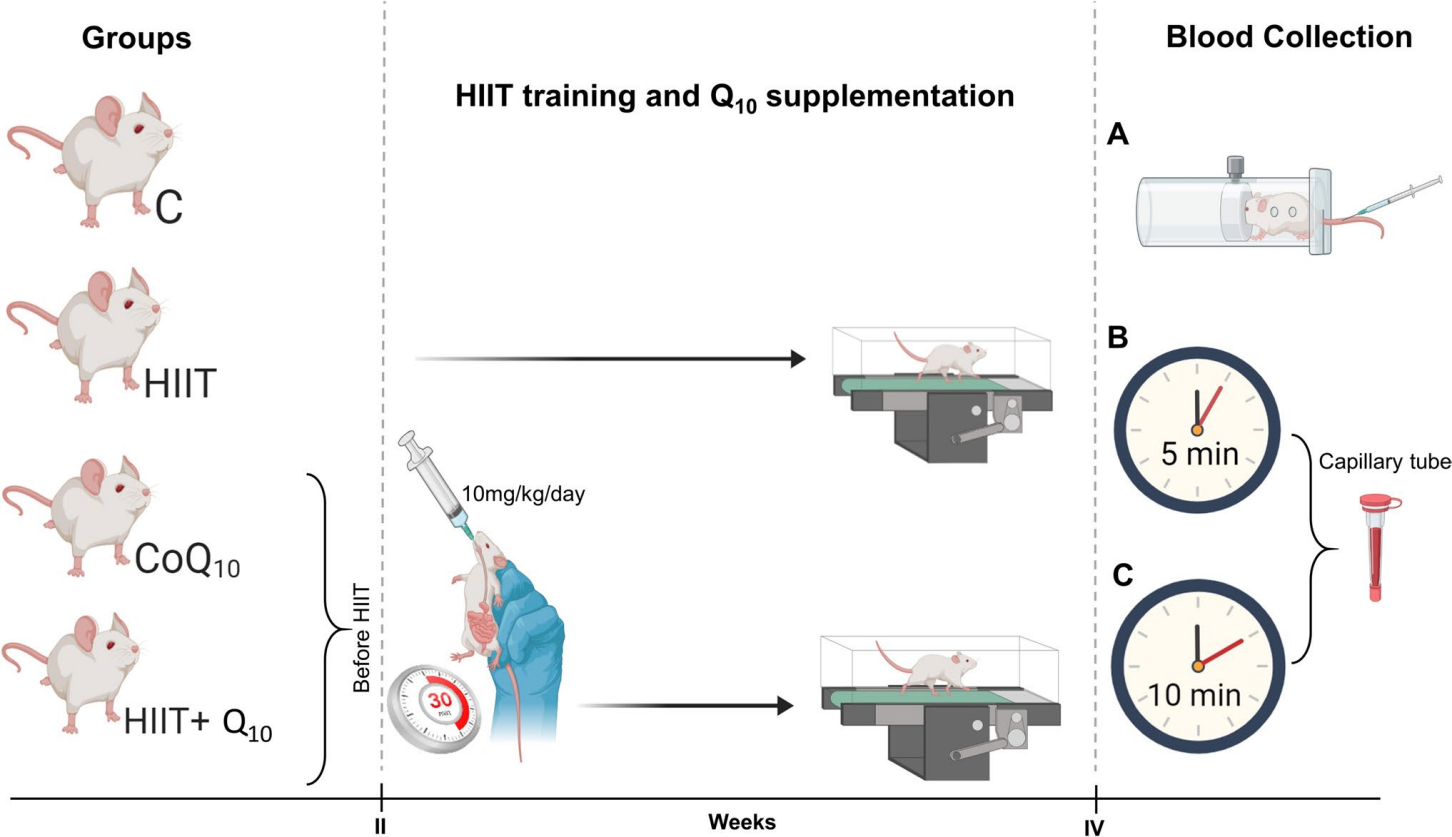
Presents a schematic representation of the experimental protocol, illustrating the timing of Q10 supplementation, HIIT interventions, and blood collection at weeks I, II, III, and IV. *C* Control group, *HIIT* High-Intensity Interval Training, *Q*_*10*_ Coenzyme Q_10_ supplement, **A** Blood collection from the tail of rats using a special device, **B** Observation time; 5 min, blood sample collected in a capillary tube, **C** Observation time; 10 min, blood sample collected in a capillary tube

The study was conducted using a randomized experimental controlled design with 24 male Sprague Dawley rats, aged 8 weeks and weighing 250.4 ± 6.1 gr. The research was carried out at the Gazi University Laboratory Animal Breeding and Experimental Research Center in compliance with the National Institutes of Health (NIH) guidelines for the care and use of laboratory animals. The research protocol was approved by the Gazi University Animal Experiments Ethics Committee on April 5, 2024 (Decision no: E-66332047-604.01-923246) and was conducted in accordance with the principles of the Helsinki Declaration, ensuring animal rights and welfare were upheld. According to G-power analysis, with a Type I error (alpha) of 0.05, a power (1-beta) of 0.8, an effect size of 1.88, and a two-tailed alternative hypothesis (H1), the minimum sample size required to detect a significant difference using this test was determined to be at least six rats per group. Consequently, the rats were randomly divided into four groups: control group (C), coenzyme Q_10_ group (CoQ_10_), HIIT group, and HIIT + Q_10_ group. The rats were housed in standard cages (two rats per cage) under controlled environmental conditions of 22–25 °C, with a 12-h light/12-h dark cycle, and were fed ad libitum. The C group was fed ad libitum without supplementation or exercise. The exercise groups underwent a four-stage preparatory phase before receiving supplementation and HIIT trials. The rats completed four stages of training: treadmill acclimatization in the first stage (KN-73; Natsume Seisakusho Co., Ltd., Tokyo, Japan), moderate-intensity continuous training (MICT) acclimatization in the second stage, high-intensity continuous training (HICT) acclimatization in the third stage (Soya et al. 2007; Yasul et al. 2025), and HIIT acclimatization in the fourth stage (Okamoto et al. 2021). The structured progression aligns with previous findings that emphasize its role in enhancing physiological resilience and optimizing adaptation to high-intensity exercise. During the I week of HIIT trials, only exercise was performed for the HIIT group and the HIIT + Q_10_ group. In the II week, the HIIT group continued with exercise only, while the HIIT + Q_10_ and CoQ_10_ groups received a 10 mg/kg/day Q_10_ supplement 30 min before exercise via gavage, a timing selected based on its pharmacokinetics, ensuring peak plasma levels coincide with the exercise period to enhance mitochondrial function and metabolic efficiency. This protocol was repeated in the III and IV weeks. The HIIT protocol was designed based on the principle of progressive overload, with the exercise session scheduled at 10:30 AM (Table 1). Additionally, during the HIIT trials weeks, blood samples were collected using capillary tubes at the fifth (first observation time) and tenth minutes (second observation time) after exercise (Fig. 1), as these time points capture early-phase metabolic recovery, reflecting rapid lactate threshold and blood gas stabilization, which are critical for evaluating post-exercise physiological responses. The blood gas values (pH, pCO_2_, pO_2_), oximetry values (Hb, HTC, sO_2_, COHb, HHb, MetHb), metabolite values (Lac, Glu), temperature corrected values (pH (T), pCO_2_ (T), pO_2_ (T)), oxygen status (p50, O_2_), acid base status (Base (Ecf), Base (B), HCO_2_- (st), HCO_2_-), electrolyte values (K + , Na + , Ca +) markers were evaluated and blood gas analyses were performed using the Radiometer 900.

**Table 1:** presents a structured high-intensity interval training (HIIT) protocol that includes a pre-training phase, progressive training phases, and a comparison of conditions with and without Coenzyme Q10 (Q10) supplementation

High-intensity interval training (HIIT) and HIIT + Q_10_ supplementation training protocols								
Pre-HIIT phase								
Phases	Training modality	Days	Reps	Sets	Running speed	Exercise duration	Rest between reps. (sec/min)	Rest between sets (min/hour)
	Treadmill Drills	0–5	1	–	6–10 (m/min)	8–12 min/hour	–	–
Phase 1	MICT Drills	6–10	1	–	11–15 (m/min)	14–20 min/hour	–	–
Phase 2	HICT Drills	11–15	1	–	18–25 (m/min)	14–20 min/hour	–	–
Phase 3	HIIT Drills	16–20	2	2	25–35 (m/min)	20 s/min	80–100	3–4
HIIT training without Q_10_ supplementation								
Week I	HIIT	21–26	2	3	30–40 (m/min)	30 s/min	120–150	3–4
HIIT Training with Q_10_ Supplementation								
Week II	HIIT	27–32	3	3	30–40 (m/min)	30 s/min	120–150	3–4
Week III	HIIT	33–38	4	4	30–40 (m/min)	30 s/min	120–150	3–4
Week IV	HIIT	39–44	5	4	30–40 (m/min)	30 s/min	120–150	3–4

Training intensity progressively increases over four distinct phases before transitioning into the main HIIT intervention. Rest intervals are systematically adjusted to optimize recovery and adaptation. The supplementation phase begins from Week II, allowing direct comparisons between supplemented and non-supplemented conditions

presents the characteristics of exercise intensity for the two groups undergoing high-intensity interval training

*HIIT* high-intensity interval training group, *HIIT + Q*_*10*_ High-intensity interval training + coenzyme Q_10_ supplementation group, *MICT* moderate-intensity continuous training, *HICT* High-intensity continuous training

### Collection ve analysis for blood gases

First, the rats were placed in a special movement-restraining device, and their tails were immersed in 40 °C warm water for 5–10 s (Parasuraman et al. 2010) to facilitate vasodilation and minimize potential discomfort during sampling. After vasodilation and the visualization of the veins, approximately 100 μl of blood was collected by inserting a 20–22G needle attached to a syringe into the ventral tail artery at an angle of 20–30 degrees, ensuring minimal distress. The collected blood samples were transferred into capillary tubes (Safeclinitubes; 942-896-D957P-70-100) and analyzed for blood gases using a Radiometer 900 autoanalyzer. To prevent continued bleeding from the tail artery, pressure was applied for a short duration, and the rats were then returned to their cages (Namlı et al. 2023). Blood sampling was performed in a controlled, quiet environment to further limit external stressors and ensure the reliability of physiological measurements.

### Statistical analysis

The study was conducted using a factorial experimental design in randomized blocks with six replications, allowing for the assessment of both main effects and interaction effects. Factorial ANOVA was performed to analyze the effects of group (C, CoQ_10_, HIIT, HIIT + Q_10_), time (5 min, 10 min), and week (I–IV) at both individual and interaction levels. The data were subjected to variance analysis (ANOVA) using JMP 13.0 software, and significant differences between means were identified using the LSD (Least Significant Difference) multiple comparison test, which was applied only when significant main or interaction effects were detected. This approach ensured a comprehensive evaluation of the combined influence of Q_10_ supplementation and HIIT on physiological parameters. Relationships between parameters were analyzed using Pearson’s correlation analysis, while multivariate relationships and scaling between treatments and physiological parameters were explored using PCA-Biplot representation, facilitated by XLSTAT (Kurt 2023). The first two principal components accounted for 67.61% of the total variance, with eigenvalues greater than 1.0, justifying their retention for analysis. This allowed for a clearer interpretation of key physiological parameters that differentiated between experimental groups.

## Results

### Blood gas values

When examining the effects of Q_10_ supplementation under HIIT conditions on pH, pCO_2_, and pO_2_ levels, varying degrees of significance were identified in terms of interventions and their interactions. All three parameters were significantly influenced by groups (G), weeks (W), and their two-way interaction (G × W), as well as by the G × M interaction for pO_2_, with at least a significance level of p < 0.05. While the time had no significant effect on the changes, the three-way interaction (G × W × M) significantly affected the pH value at the *p* < 0.05 level (Table SM-1). The mean pH value, which was 7.36 mmHg, reached its highest level of 7.418 mmHg in the HIIT + Q_10_ group during the second observation time of the IV week. However, when only groups were considered, the C group and the HIIT + Q_10_ group were statistically classified in the same group, with the lowest pH value observed exclusively in the HIIT group at 7.34 mmHg. The average pCO_2_ level, determined to be 46.05 mmHg, reached its highest value (51.48 mmHg) in the III week of the group CoQ_10_. Similarly, for pO_2_, the highest value (59.96 mmHg) was observed in the IV week of the group CoQ_10_. When interactions were disregarded the highest pO_2_ levels were predominantly identified in the HIIT + Q_10_ group (Table SM-6).

### Oximetry values

When examining the effect of Q_10_ supplementation under HIIT conditions on Hb, HCT, sO_2_, COHb, HHb, and MetHb values, significant differences were identified at various levels regarding the interventions and their interactions. For all parameters, the groups, weeks, and their two-way interaction (G × W) showed at least a significance level of *p* < 0.05. However, weeks were non-significant for sO_2_, and groups were non-significant for HHb. Except for MetHb, time (M) had no significant effect on the changes, but the three-way interaction (G × W × M) significantly influenced HCT levels at *p* < 0.05 (Tables SM-1, 2) (Tables SM-1, 2). For Hb, with an overall mean value of 14.68 g/dl, the highest level, 16.36 g/dl, was reached in the III week in the HIIT + Q_10_ group. When only groups were considered, the HIIT + Q_10_ group had the highest Hb level at 15.22 g/dl. Similarly, for HCT, which had an overall mean of 44.54, the HIIT + Q_10_ group recorded the highest value (47.19), while the C group had the lowest value (41.92). In the two-way interaction (G × W), the HIIT + Q_10_ group exhibited the highest HCT value (50.13), while the C group showed the lowest. In the three-way interaction (G × W × M), particularly during the second observation time in the II week of supplementation, the HIIT + Q_10_ group demonstrated significant increases. The highest Hb value, 15.22 g/dl, was identified in the HIIT + Q_10_ group. For sO_2_, the CoQ_10_ group, HIIT group, and HIIT + Q_10_ group belonged to the same statistical category, while the lowest value, %50.41, was observed in the C group. Regarding COHb, which had an overall mean of %1.45, the lowest value, %1.07, was statistically distinct and observed in the HIIT + Q_10_ group. In the G × W interaction, COHb reached its highest level during the I week in the HIIT group (3.02) and the II week in the CoQ_10_ group (2.81). The lowest COHb value, %0.53, was recorded in the III week in the HIIT + Q_10_ group. For HHb, which had an overall mean of %43.65, the highest values were observed in the II (%46.12) and III (%47.42) weeks, while the lowest value occurred in the IV week (%38.68). MetHb, with an overall mean of %0.42, reached its highest value in the IV week in the CoQ_10_ group (%1.17). Among the groups, the HIIT group recorded the lowest MetHb value at %0.16 (Table SM-7).

### Metabolite values

**Fig. 2:**
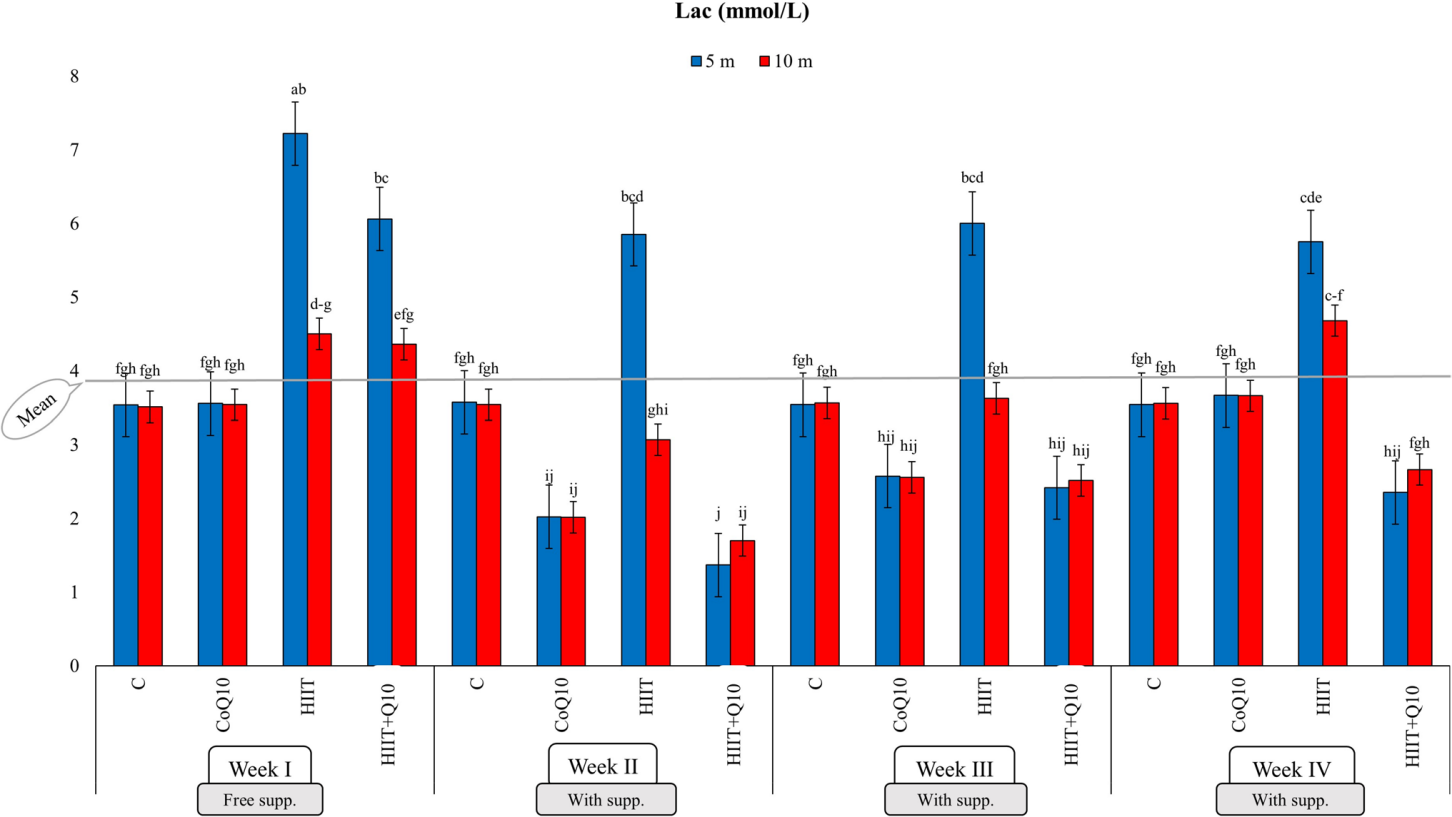
Four-week follow-up of the lactate (Lac) mmol/L value at two different observation times. *Lac* Lactate concentration in blood, C Control group, *CoQ*_*10*_ Coenzyme Q_10_ supplement group, *HIIT* High-Intensity Interval Training group, *HIIT + Q*_*10*_ High-Intensity Interval Training + Coenzyme Q_10_ supplement group, 5 m: 5th minute (first observation time), 10 m: 10th minute (second observation time), the letters above the columns (a, b, c, d, e, f, g, h, j) indicate statistical differences between groups analyzed by the One-Way ANOVA test followed by the LSD multiple comparison test

The effect of Q_10_ supplementation under HIIT conditions on Lac was significant at the *p* < 0.01 level for all factor and their two-way and three-way interactions. For Glu, significance was observed in G, W, G × W, and G × W × M interactions (Tables SM-2, 3). The average Lac value, calculated as 3.72 mmol/L, reached its highest level of 7.76 mmol/L in the HIIT group during the first observation time in the III week. However, the HIIT + Q_10_ group had the lowest Lac level at the same observation time, with a value of 2.42 mmol/L. At the second observation time in the IV week, the HIIT group exhibited the highest Lac value at 4.68 mmol/L, while the HIIT + Q_10_ group recorded the lowest Lac value during the second observation time in the II week. Furthermore, when only the groups were compared, the HIIT + Q_10_ group and the CoQ_10_ group were found to belong to the same statistical category, while the highest Lac value, 5.33 mmol/L, was exclusively observed in the HIIT group. Regarding weeks, the Lac level, which was at its peak during the I week, declined in subsequent weeks following supplementation intervention (Fig. 2). As for Glu, the overall study average was 186.24 mg/dL, with the CoQ_10_ group reaching the highest value (259.68 mg/dL) in the IV week. Among the groups, the highest Glu value (205.71 mg/dL) was identified in the HIIT + Q_10_ group (Table SM-8).

### Temperature corrected values

When examining the effect of Q_10_ supplementation under HIIT conditions on pH (T), pCO_2_ (T), and pO_2_ (T) values, varying levels of significance were identified in terms of the interventions and their interactions. Among these, the parameters pH (T), pCO_2_ (T), and pO_2_ (T) showed significant differences between groups and weeks, as well as in their interaction (G × W) at the *p* < 0.01 level, while pCO_2_ (T) was influenced by weeks at the *p* < 0.05 level (Table SM-3). The mean pH (T) value, recorded at 7.36, reached its highest level of 7.40 in the IV week in the HIIT + Q_10_ group. When only groups were considered, the CoQ_10_ group, HIIT group, and HIIT + Q_10_ group were found to belong to the same statistical group, with the highest pH (T) value observed exclusively in the C group at 7.38 mmHg. In terms of weeks, the lowest pH (T) value was observed in the I week, while the highest was recorded in the IV week. The mean pCO_2_ (T) value, calculated at 45.95, was found to be highest in the CoQ_10_ group (47.64) when only groups were analyzed. In terms of weeks, the highest pCO_2_ (T) value was observed in the III week. Regarding the interaction (G × W), the HIIT group (48.82) and the HIIT + Q_10_ group (46.67) were above the overall mean in the I week, while the HIIT + Q_10_ group was below the mean in the II and III weeks. The mean pO_2_ (T) value, recorded at 46.94, showed the highest value in the HIIT group (51.15) when only groups were considered. In terms of weeks, the highest pO2 (T) value was observed in the IV week (Table SM-9).

### Oxygen status

The effects of Q_10_ supplementation under HIIT conditions on p50 and O_2_ (vol%) values were analyzed, revealing significant differences based on G, W, M, and their interactions. The p50 value was significantly influenced by G, W, T, and pairwise interactions (G × W and G × M) at *p* < 0.01, and by the three-way interaction (G × W × M) at *p* < 0.05 (Table SM-3,4). The mean p50 value was 37.35 mmHg, with the highest value (41.71 mmHg) recorded in the HIIT + Q_10_ group during the first observation time of the I week. Among the groups, the lowest p50 value (35.27 mmHg) was observed in the C group. The O_2_ (vol%) value was significantly affected by G and W at *p* < 0.05 and by pairwise interactions (G × W) at *p* < 0.01. The mean O_2_ (vol%) value was 12.40, with the lowest value (11.11) observed in the C group and the highest value (13.65) recorded in the IV week. The CoQ_10_ group and HIIT + Q_10_ group reached the highest O_2_ (vol%) values during the IV week, placing them in the same statistical group (Table SM-10).

### Acid–base status values

The effects of Q_10_ supplementation on metabolic parameters under HIIT conditions were evaluated. When examining Base (Ecf), Base (B), HCO_3_- (st), and HCO_3_- values, statistically significant differences were identified in terms of interventions and interactions at least at the *p* < 0.05 and *p* < 0.01 levels (Table SM-4). The overall mean of HCO_3_- (st) was 23.38 mmol/L, showing an increase from the I week and reaching its peak level. In the HIIT + Q_10_ group, the lowest value was recorded in the I week, followed by an increase in subsequent weeks. The mean Base (Ecf) value was 0.67 mmol/L, with the negative value in the I week transitioning to a positive increase. The lowest positive value was observed in the HIIT + Q_10_ group, while the highest was recorded in the CoQ_10_ group. The mean Base (B) value was 0.13 mmol/L, where the initially negative value in the I week turned positive in subsequent weeks. The mean HCO_3_- value was calculated as 25.51 mmol/L, with the maximum level reached in the IV week in the HIIT + Q_10_ group (Table SM-11).

### Electrolyte values

When examining the effects of Q_10_ supplementation under HIIT conditions on K^+^, Na^+^, and Ca^+^ levels, significant differences were identified at various levels in terms of treatments and their interactions. The K^+^ and Ca^+^ levels were influenced by G, W, and their two-way interaction (G × W), with Ca^+^ also being affected by the interaction (G × M), and K^+^ by the interaction (W × M), all at a significance level of at least *p* < 0.05. The Na^+^ level was significantly influenced by G and W (*p* < 0.05) and by two-way interactions (G × W and W × M) at a significance level of at least *p* < 0.01 (Table SM-5). The mean K^+^ level, recorded as 3.96 mmol/L, showed that the HIIT group and the HIIT + Q_10_ group were within the same statistical category, with the highest K^+^ level observed in the C group at 4.64 mmol/L. The Na^+^ level, with an overall mean of 142.46 mmol/L, was lowest in the HIIT group at 141.47 mmol/L. For Ca^+^, the highest level (1.42 mmol/L) was observed in the C group, while the lowest level (1.32 mmol/L) was found in the HIIT + Q_10_ group. At the second observation time, the HIIT group and HIIT + Q_10_ group were within the same statistical category, although the Ca^+^ level in the HIIT + Q_10_ group was lowest (1.33 mmol/L) at the first observation time (Table SM-12).

### Correlation

**Fig. 3:**
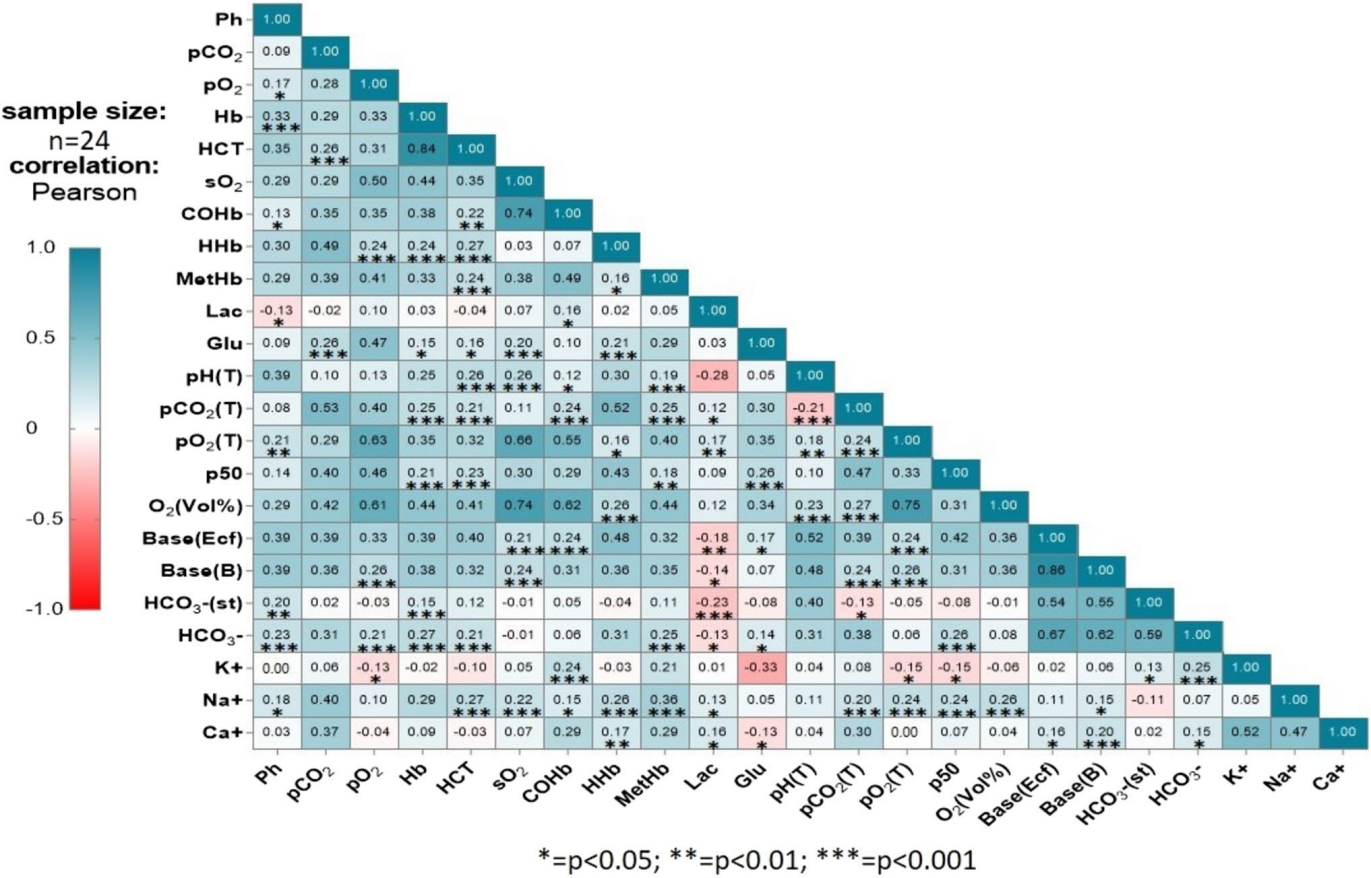
Correlation between Lac and other blood gas measurements using Pearson correlation analysis. *pCO*_*2*_ Partial pressure of carbon dioxide, *pO*_*2*_ partial pressure of oxygen, *Hb* hemoglobin, *HCT* hematocrit, *sO*_*2*_ Oxygen saturation, *COHb* Carboxyhemoglobin, *HHb* Deoxyhemoglobin, *MetHb* Methemoglobin, *Lac* Lactate, *Glu* Glucose, *O2(vol%)* Oxygen concentration, *Base(Ecf)* Base excess in extracellular fluid, *Base(B)* Base excess in blood, *HCO*_*3*_*-(st)* Standard bicarbonate, *HCO*_*3*_*-* Bicarbonate, *K*^*+*^ Potassium, *Na*^*+*^ Sodium, *Ca*^*+*^ Calcium

The results of the Pearson correlation analysis demonstrated statistically significant but low-level positive and negative correlations among biochemical parameters. Specifically, weak positive or negative correlations were identified among pH, Na^+^, HCO_3_⁻, Lac, COHb, Hb, and pO_2_, while pCO_2_ was found to be associated with Glu and HTC. Hb exhibited correlations with HCO_3_⁻ and HHb, whereas HTC showed low-level positive relationships with parameters such as Na^+^, HCO_3_⁻, and MetHb. Positive correlations were detected between Lac and Ca^+^, while negative correlations were observed between Lac and HCO_3_⁻ or pH. Additionally, Glu exhibited a negative correlation with Ca^+^ and a positive correlation with p50. pO_3_ was positively associated with Na^+^, Base(B), and Base(Ecf), while significant relationships were identified between pCO_2_(T) and O_2_(Vol%). These findings are illustrated in Fig. 3.

**Fig. 4:**
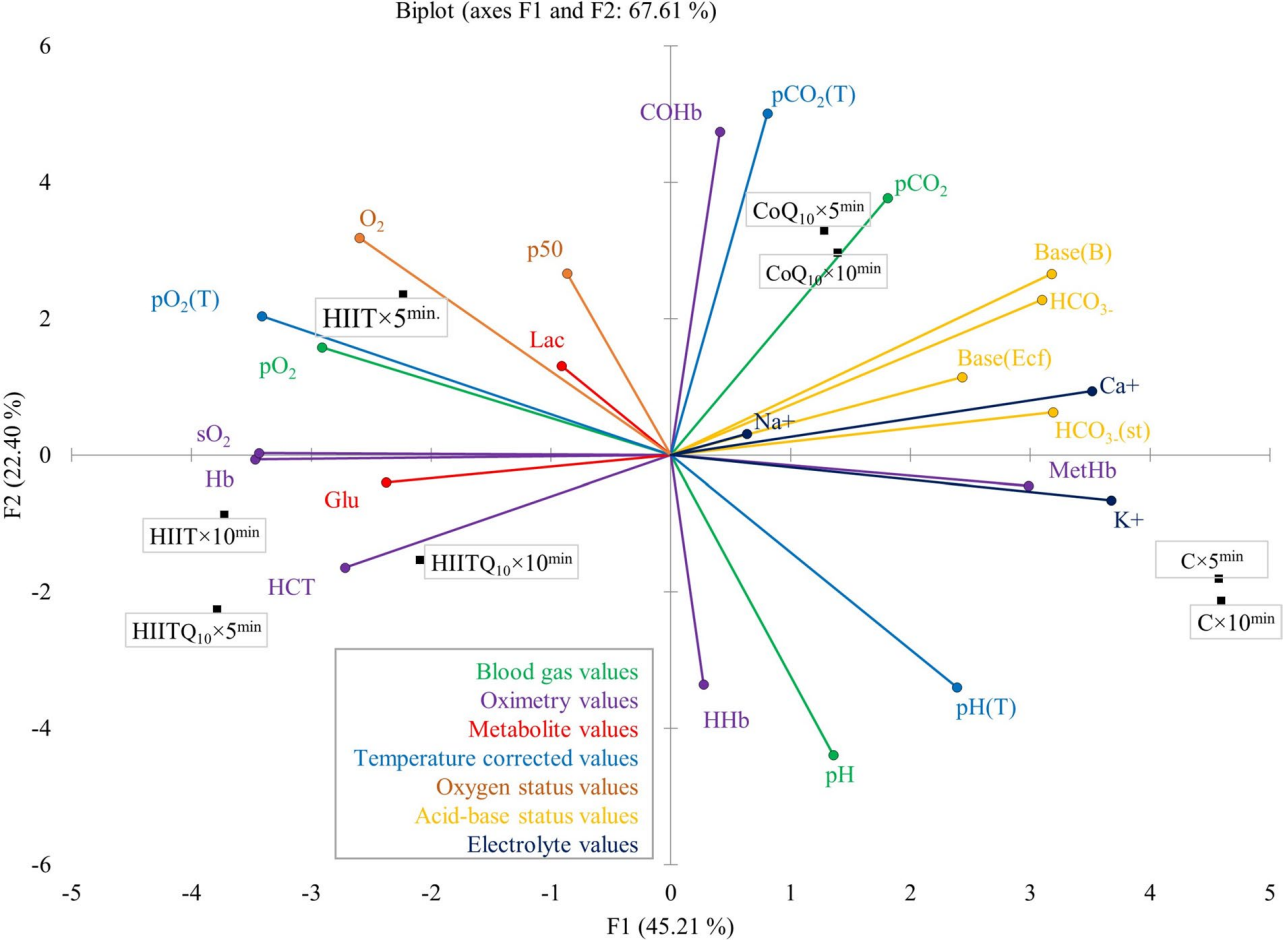
Responses of different parameters to changing conditions, differences between groups, and relationships between parameters using principal component analysis (PCA). *pCO*_*2*_ partial pressure of carbon dioxide, *pO*_*2*_ partial pressure of oxygen, *Hb* hemoglobin, *HCT* hematocrit, *sO*_*2*_ oxygen saturation, *COHb* carboxyhemoglobin, *HHb* deoxyhemoglobin, *MetHb* methemoglobin, *Lac* lactate, *Glu* glucose, *O2(vol%)* oxygen concentration, *Base(Ecf)* base excess in extracellular fluid, *Base(B)* base excess in blood, *HCO*_*3*_*-(st)* Standard bicarbonate, *HCO*_*3*_*-* bicarbonate, *K*^*+*^ potassium, *Na*^*+*^ sodium, *Ca*^*+*^ calcium

Principal component analysis (PCA) was conducted to determine the relative importance of the parameters and their contributions to total variance. The first two components accounted for 67.61% of the total variance, while the first five components, with eigenvalues ≥ 1, explained 99.39% of the variance. The largest contributions to the F1 component were made by K^+^ (9.1%) and Ca^+^ (8.3%), followed by Hb, sO_2_, pO_2_(T), HCO_3_⁻(st), and cBase(B). For the F2 component, the highest contributions were from HTC (16.9%), pH (15.2%), and HHb (13.1%), followed by CoHb, O_2_, pCO_2_, and MetHb. The PCA biplot graph revealed the adaptive capacity of parameters to variable conditions. The parameters located near the origin represented higher adaptability, while Na^+^, Lac, p50, and Glu emerged as significant parameters under HIIT and Q_10_ interventions. Notably, Na^+^ levels were more stable in the CoQ_10_ groups, suggesting a potential role in maintaining electrolyte balance during exercise stress. Meanwhile, Lac levels were significantly lower in the HIIT + Q_10_ group compared to the HIIT group, reinforcing the hypothesis that Q_10_ enhances lactate clearance and aerobic energy production. Similarly, Glu levels were highest in the HIIT + Q_10_ group, indicating enhanced glucose utilization and energy availability, possibly due to Q_10_'s role in mitochondrial efficiency. A strong relationship between the HIIT + Q_10_ group and HTC was evident, while pCO_2_ demonstrated a notable association with the CoQ_10_ group. In the HIIT-only group, O_2_ and Lac were prominent in the early phase, whereas Glu dominated in the later phase. This suggests that O_2_ emerged as a key parameter in the HIIT + Q_10_ group. The vector lengths and angles in the PCA biplot indicated relationships between parameters: angles smaller than 90° indicated positive correlations, while those greater than 90° signified negative correlations. For instance, a strong negative correlation was found between pO_2_ and pH, whereas no significant association was observed with pCO_2_. Variations in the responses of parameters within the same group were inferred from the tendency of vectors to diverge from or converge toward the center. These findings suggest that CoQ_10_ supplementation may contribute to metabolic optimization during high-intensity exercise, a trend that was clearly visualized through PCA group separation. Notably, MetHb and K^+^ were positioned near the C group, whereas pH(T) and HHb distanced themselves from the HIIT and Q_10_ factors, occupying distinct regions. The correlation findings indicate that biochemical balance is shaped by multiple factors and that Q_10_ supplementation, in combination with HIIT exercise, significantly contributes to physiological adaptation processes (Fig. 4).

## Discussion

Under HIIT conditions, it was observed that lactate threshold levels showed a significant reduction in the HIIT + Q_10_ group receiving Q_10_ supplementation, with these levels remaining below average. Additionally, the combination of Q_10_ supplementation with HIIT resulted in significant improvements in blood gas parameters (pH, pO_2_, and pCO_2_) and enhanced oxygen transport capacity, as indicated by oximetry parameters (Hb, HCT, and sO_2_). Moreover, the reduction in COHb levels highlighted Q_10_ supplementation as an effective strategy for alleviating exercise load. From an acid–base balance perspective, positive changes in HCO_3_- and base values contributed to a reduction in metabolic stress, with this effect becoming more pronounced over time. Regarding electrolyte balance, regulatory effects were particularly noted in K^+^ and Na^+^ levels. These findings demonstrate that the integration of Q_10_ supplementation with HIIT provides significant benefits on both physiological and metabolic parameters.

The homeostasis of H^+^ ions in body fluids and cells is crucial for maintaining healthy cellular biological processes and keeping the pH levels of intracellular and extracellular fluids within narrow limits (Gençoğlu et al. 2022). High-intensity exercises promote Lac accumulation, triggering H^+^ ion release in muscles, which disrupts the body’s acid–base homeostasis. This condition shifts pH levels toward acidosis (Fosstveit et al. 2024). In current study, the HIIT group exhibited significant Lac accumulation and lower pH levels at the first observation time during the I week compared to the C. However, by the second observation time, partial improvement in buffering capacity had brought pH levels to an acceptable range. Cao et al. (2021) reported that prolonged HIIT in rats stimulated lactate production in muscles, increasing blood lactic acid levels. This created an acidic microenvironment, rapidly lowering pH levels, and, when buffering capacity was insufficient, negatively impacted performance. Thus, when HIIT is performed alone, it disrupts acid–base balance and increases metabolic stress. This situation indicates increased metabolic acidosis associated with impaired acid–base balance during HIIT (Lühker et al. 2018). High-intensity exercises involving the activation of large muscle groups, such as running, negatively affect acid–base parameters like arterial pH, Base(Ecf), Base(B), HCO_3_-(st), and HCO_3_- in a short period (Lindinger and Heigenhauser 2012). However, from the second week onward, Q_10_ supplementation mitigated these effects by significantly limiting Lac accumulation and enhancing buffering capacity in the HIIT + Q_10_ group. Specifically, Base(Ecf), Base(B), HCO_3_-(st), and HCO_3_- values indicated better maintenance of acid–base balance, with notable improvements in recovery during the HIIT + Q_10_ group. By the fourth week, the HIIT + Q_10_ group achieved the highest positive values in metabolic parameters, displaying a profile of effectively managed oxidative stress. These results highlight the beneficial contributions of coenzyme Q_10_ supplementation in balancing oxidative stress, improving metabolic parameters, and supporting post-exercise performance. This relationship was also reflected in the correlation analysis results.

Broome et al. (2021) provided evidence showing that well-trained middle-aged male cyclists exhibited better time performance and higher power output in an 8-km time trial after 28 days of coenzyme Q_10_ supplementation compared to a placebo group. Similarly, Hostrup et al. (2023) reported that 6 weeks of HIIT, performed three times per week by nine young men, reduced K^+^ levels in skeletal muscle. In current study, the parameters such as K^+^, Ca^+^, HCT, pH, and HHb played a negative role in performance in the HIIT group. However, the differentiation of the HIIT + Q_10_ group from other groups suggested that Q_10_ supplementation provided a biological advantage and improved performance within this group. Notably, the significant reduction in Ca^+^ levels observed in the HIIT + Q_10_ group was statistically significant, suggesting a potential regulatory role of Q_10_ in calcium homeostasis. This aligns with the findings of Petrofsky et al. (2012), who suggested that Q_10_ stabilizes calcium channels indirectly, reducing intracellular calcium imbalance. Additionally, Dos Santos et al. (2024) linked decreased serum Ca^+^ levels after eight weeks of HIIT to an increase in myofilament sensitivity to Ca^+^, which may enhance cardiomyocyte contractile function. These findings indicate that the observed Ca^+^ reduction in the HIIT + Q_10_ group may contribute to improved muscle contractility and fatigue resistance, further supporting the role of Q_10_ in performance enhancement.

The balanced pCO_2_ values observed in the HIIT + Q_10_ group compared to other groups indicated that Q_10_ supplementation plays a stabilizing role under the strenuous conditions of HIIT, enhancing both performance and metabolic balance. This role was further supported by the positive relationship between the HIIT + Q_10_ group and HCT. At the first observation time point, high oxygen demand and elevated lactate levels negatively affected performance, while high glucose 2 demand was a significant limiting factor at the second observation point. Emami et al. (2018), investigating the effects of Q_10_ concentration during intense physical exercise in elite swimmers, emphasized that a 14-day Q_10_ supplementation acted as a cellular stabilizer, regulated core body temperature, and served as a crucial cofactor in oxidative phosphorylation, thereby increasing the time to exhaustion associated with fatigue. In current study, Q_10_ supplementation and HIIT interventions influenced pH(T), pCO_2_(T), and pO_2_(T) values differently across groups and weeks. While Q_10_ supplementation alone in the CoQ_10_ group showed limited effects, the HIIT + Q_10_ group positively impacted both performance and metabolic balance.

One of the key processes for performance enhancement is glucose-driven ATP production. Research exploring the effects of HIIT on energy metabolism consistently reported significant reductions in glucose levels (Flockhart et al. 2023; Chen et al. 2024). Additionally, Q_10_ supplementation has been shown to increase 2,3-diphosphoglycerate levels in erythrocytes, facilitating pO_2_ transfer at specific pressures. This mechanism not only enhances oxygen delivery to skeletal muscles but also improves oxygen availability in cardiac and respiratory muscles, potentially boosting ATP synthesis, optimizing glucose utilization, reducing lactate production, and increasing VO_2_max (Zheng and Moritani 2008). In current study, Q_10_ supplementation was found to significantly influence Glu levels, with the highest Glu average observed in the HIIT + Q_10_ group. This suggests a synergistic effect of Q_10_ supplementation and HIIT in regulating energy metabolism and glucose levels, thereby enhancing time to exhaustion and performance output.

## Conclusion

The Q_10_ supplementation has been recognized for its effects on metabolic balance and performance during HIIT training. The supplementation provided significant improvements, particularly in pH and pO_2_ values, while contributing to the balanced maintenance of pCO_2_ levels in the HIIT + Q_10_ group. Lactate (Lac) levels remained high in the HIIT group but showed a marked reduction in the HIIT + Q_10_ group. Glucose (Glu) levels, on the other hand, reached their highest values (205.71 mg/dL) in the HIIT + Q_10_ group. These findings reveal the potential of Q_10_ supplementation to reduce metabolic stress and support energy metabolism. In terms of oximetry parameters, Q_10_ supplementation enhanced oxygen transport capacity (Hb, HCT, sO_2_) and reduced COHb levels, thereby alleviating exercise load. Additionally, it demonstrated positive effects on acid–base balance by reducing metabolic load, as indicated by increases in HCO_3_- and base levels. Regarding electrolyte levels, the stabilizing effects on K^+^ and Na^+^ were notable, while changes in Ca^+^ levels were identified as an important parameter for further investigation. PCA analyses revealed that Q_10_ supplementation distinctly separated the HIIT + Q_10_ group from the others, providing a performance advantage. Pearson correlation analysis identified low but significant relationships among biochemical parameters, further supporting the contribution of Q_10_ supplementation to biochemical adaptation processes. In conclusion, the Q_10_ supplementation supported metabolic balance, improved oxygen transport, and stabilized acid–base levels during HIIT. It reduced lactate accumulation, enhanced glucose availability, and alleviated metabolic stress, thereby improving recovery efficiency and physiological adaptation.

### Strengths and limitations

This study’s strengths include its use of a randomized controlled design, a statistically powered sample size, and advanced statistical methods (PCA and Pearson’s correlation analysis), which enhance the reliability of the findings. The well-structured exercise protocol, incorporating progressive overload and acclimatization, ensures consistency, while the use of advanced equipment, such as the Radiometer 900, strengthens the precision of the results. However, the study has several limitations that must be acknowledged. First, although the sample size was statistically sufficient, it remains relatively small compared to similar preclinical studies, potentially affecting the generalizability of the findings. Second, the controlled laboratory setting provided a standardized environment; however, it does not fully replicate the complex physiological and psychological stressors encountered in real-world athletic or clinical settings. Future studies should consider incorporating additional environmental factors to improve external validity. Third, the exclusive use of male Sprague–Dawley rats limits the applicability of these results to female subjects, who may exhibit different physiological responses to HIIT and CoQ_10_ supplementation due to hormonal and metabolic variations. To address this, further research is needed to investigate potential sex-based differences and their impact on exercise-induced metabolic adaptations. Additionally, while the short study duration allowed for focused analysis, longitudinal studies are needed to examine long-term physiological adaptations to HIIT and CoQ_10_ supplementation. Finally, the reliance on an animal model, while beneficial for mechanistic insights, necessitates caution when extrapolating findings to human populations. These limitations should be addressed in future research to enhance the applicability and external validity of the results.

## Supplementary Information

Below is the link to the electronic supplementary material.

Supplementary file1 (DOCX 131 KB)

## Abbreviations

Q_10_: Coenzim Q_10_
MICT: Moderate-intensity continuous training
HICT: High-intensity continuous training
HIIT: High-intensity interval training
pCO_2_: Partial pressure of carbon dioxide
pO_2_: Partial pressure of oxygen
Hb: Hemoglobin
HCT: Hematocrit
sO_2_: Oxygen saturation
COHb: Carboxyhemoglobin
HHb: Deoxyhemoglobin
MetHb: Methemoglobin
Lac: Lactate threshold
Glu: Glucose
O_2_ (vol%): Oxygen concentration
Base(Ecf): Base status in extracellular fluid
Base(B): Base Status in blood
HCO_3_- (st): Standard bicarbonate
HCO_3_-: Bicarbonate
K^+^: Potassium
Na^+^: Sodium
Ca^+^: Calcium

## Declarations

### Data availability

The corresponding author is available to provide the primary data upon request.
