# Supplementary material for: Evaluating the impact of coenzyme Q10 and high-intensity interval training on lactate threshold and Plasma blood gases in rats: a randomized controlled trial
Source: Eur J Appl Physiol. 2025 Mar 18;125(8):2185–96. doi: 10.1007/s00421-025-05756-8 (PMC12354119; doi:10.1007/s00421-025-05756-8)
Supplement: Supplementary file 1 — Supplementary file1 (DOCX 131 KB) [file 421_2025_5756_MOESM1_ESM.docx]

**SUPPLEMENTARY MATERIALS**

**Full Title: Evaluating the Impact of Coenzyme Q10 and High-Intensity Interval Training on Lactate Threshold and Plasma Blood Gases in Rats: A Randomized Controlled Trial**

**Authors:** Yavuz Yasul^1^, Büşra Yılmaz^2^, Ömer Şenel^2^, Dursun Kurt^1^, Taner Akbulut^3^, Ayşen Çalıkuşu^4^, Elvan Anadol^5^, Canan Yılmaz^6^

**Affiliations:**

^1^Ondokuz Mayıs University, Bafra Vocational School, Samsun, Turkiye.

^2^Gazi University, Faculty of Sport Sciences, Ankara, Turkiye.

^3^Fırat University, Faculty of Sport Sciences, Elazığ, Turkiye.

^4^Ege University, Department of Anatomy, Izmir, Turkiye.

^5^Gazi University, Laboratory Animals Breeding and Experimental Researches Center, Ankara, Turkiye.

^6^Gazi University, Faculty of Medicine, Department of Medical Biochemistry, Ankara, Turkiye.

**Address for Correspondence**:

Yavuz YASUL, PhD

Bafra Vocational School, Ondokuz Mayıs University

Bafra Vocational School 55400 Bafra, Samsun, Turkiye

yavuz.yasul@omu.edu.tr

**Supplemental Table S1.** The ANOVA table for the significance of treatments and their interactions according to factorial experiment design in randomized blocks for pH, pCO_2_, pO_2_, Hb and HCT.

| **Sources** |  | **pH** | | | **pCO_2_ (mmHg)** | | | | **pO_2_ (mmHg)** | | | | **Hb (g/dl)** | | | **HCT (%)** | | |
| --- | --- | --- | --- | --- | --- | --- | --- | --- | --- | --- | --- | --- | --- | --- | --- | --- | --- | --- |
|  | DF | MS |  | LSD_0.05_ | MS |  | LSD_0.05_ | MS | |  | LSD_0.05_ | MS | |  | LSD_0.05_ | MS |  | LSD_0.05_ |
| Block | 5 | 0.0070 |  |  | 134.93 |  |  | 793.85 | |  |  | 17.8020 | |  |  | 92.194 |  |  |
| Group | 3 | 0.0039 | * | 0.015 | 127.32 | ** | 1.896 | 584.06 | | ** | 3.062 | 12.0303 | | ** | 4.527 | 224.37 | ** | 1.764 |
| Week | 3 | 0.0185 | ** | 0.015 | 82.963 | * | 1.896 | 659.78 | | ** | 3.062 | 11.4241 | | ** | 4.527 | 172.88 | ** | 1.764 |
| Group × Week | 9 | 0.0056 | ** | 0.030 | 44.829 | * | 3.792 | 410.97 | | ** | 6.123 | 11.5720 | | ** | 1.155 | 59.944 | ** | 3.530 |
| Minute | 1 | 0.0001 |  |  | 5.576 |  |  | 0.3300 | |  |  | 0.0024 | |  |  | 3.4534 |  |  |
| Group × Minute | 3 | 0.0008 |  |  | 4.117 |  |  | 18.555 | |  |  | 0.2641 | |  |  | 1.0180 |  |  |
| Week × Minute | 3 | 0.0014 |  |  | 12.065 |  |  | 195.63 | | * | 4.331 | 3.1254 | |  |  | 24.412 |  |  |
| Group × Week × Minute | 9 | 0.0028 | * | 0.041 | 11.388 |  |  | 76.557 | |  |  | 2.7730 | |  |  | 41.237 | * | 4.991 |
| Error | 155 | 0.0014 |  |  | 22.131 |  |  | 57.696 | |  |  | 2.0527 | |  |  | 19.167 |  |  |

DF: Degree of freedom, MS: Mean Squares, LSD: Least significant difference. *: p<0.05, **: p<0.01, pCO_2_: partial pressure of carbon dioxide, pO_2_: partial pressure of oxygen, Hb: hemoglobin, HCT: hematocrit

**Supplemental Table S2.** The ANOVA table for the significance of treatments and their interactions according to factorial experiment design in randomized blocks for sO_2_, COHb, HHb, MetHb and Lac.

| **Sources** |  | **sO_2_ (%)** | | | **COHb (%)** | | | | **HHb (%)** | | | | **MetHb (%)** | | | **Lac (mmol/L)** | | |
| --- | --- | --- | --- | --- | --- | --- | --- | --- | --- | --- | --- | --- | --- | --- | --- | --- | --- | --- |
|  | DF | MS |  | LSD_0.05_ | MS |  | LSD_0.05_ | MS | |  | LSD_0.05_ | MS | |  | LSD_0.05_ | MS |  | LSD_0.05_ |
| Block | 5 | 3068.8 |  |  | 12.844 |  |  | 1982.2 | |  |  | 1.8686 | |  |  | 0.3174 |  |  |
| Group | 3 | 589.23 | * | 5.928 | 4.4625 | * | 0.456 | 86.419 | |  |  | 1.8432 | | ** | 1.896 | 58.645 | ** | 0.258 |
| Week | 3 | 239.75 |  |  | 4.6881 | * | 0.456 | 753.29 | | * | 5.996 | 2.0979 | | ** | 1.896 | 22.175 | ** | 0.519 |
| Group × Week | 9 | 802.26 | ** | 11.854 | 5.2386 | ** | 0.833 | 565.47 | | ** | 11.09 | 0.8688 | | ** | 0.381 | 6.5534 | ** | 0.843 |
| Minute | 1 | 23.123 |  |  | 0.2041 |  |  | 118.61 | |  |  | 0.4981 | |  |  | 22.392 | ** | 0.047 |
| Group × Minute | 3 | 218.50 |  |  | 0.3554 |  |  | 84.064 | |  |  | 0.1696 | |  |  | 22.275 | ** | 0.367 |
| Week × Minute | 3 | 149.65 |  |  | 1.9279 |  |  | 105.21 | |  |  | 0.8385 | | * | 0.270 | 3.5480 | ** | 0.367 |
| Group × Week × Minute | 9 | 62.940 |  |  | 12.844 |  |  | 102.45 | |  |  | 0.3282 | |  |  | 2.1716 | ** | 7.368 |
| Error | 155 | 216.15 |  |  | 1.2847 |  |  | 221.23 | |  |  | 0.2255 | |  |  | 0.4180 |  |  |

DF: Degree of freedom, MS: Mean Squares, LSD: Least significant difference. *: p<0.05, **: p<0.01, sO_2_: oxygen saturation, COHb: [carboxyhemoglobin](https://tureng.com/tr/turkce-ingilizce/carboxyhemoglobin), HHb: deoxyhemoglobin, MetHb: methemoglobin, Lac: lactate.

**Supplemental Table S3.** The ANOVA table for the significance of treatments and their interactions according to factorial experiment design in randomized blocks for Glu, pH(T), pCO_2_(T), pO_2_(T) and p50.

| **Sources** |  | **Glu (mg/dl)** | | | **pH(T)** | | | **pCO_2_(T) (mmHg)** | | | **pO_2_(T) (mmHg)** | | | | **p50 (mmHg)** | | | |
| --- | --- | --- | --- | --- | --- | --- | --- | --- | --- | --- | --- | --- | --- | --- | --- | --- | --- | --- |
|  | DF | MS |  | LSD_0.05_ | MS |  | LSD_0.05_ | MS |  | LSD_0.05_ | MS |  | LSD_0.05_ | MS | |  | LSD_0.05_ |  |
| Block | 5 | 12019.4 |  |  | 0.0295 |  |  | 153.50 |  |  | 834.53 |  |  | 122.97 | |  |  |  |
| Group | 3 | 10660.0 | ** | 11.172 | 0.0113 | ** | 0.019 | 88.318 | ** | 1.836 | 1225.2 | ** | 4.059 | 99.305 | | ** | 1.155 |  |
| Week | 3 | 48730.2 | ** | 11.172 | 0.0245 | ** | 0.019 | 60.989 | * | 1.836 | 1269.1 | ** | 4.059 | 50.660 | | ** | 1.155 |  |
| Group × Week | 9 | 12625.0 | ** | 22.345 | 0.0152 | ** | 0.041 | 65.991 | ** | 3.673 | 342.44 | ** | 8.118 | 25.625 | | ** | 2.311 |  |
| Minute | 1 | 2389.53 |  |  | 0.0049 |  |  | 0.9130 |  |  | 48.693 |  |  | 68.116 | | ** | 0.815 |  |
| Group × Minute | 3 | 818.230 |  |  | 0.0017 |  |  | 24.743 |  |  | 72.162 |  |  | 38.408 | | ** | 1.633 |  |
| Week × Minute | 3 | 1432.02 |  |  | 0.0014 |  |  | 19.400 |  |  | 87.585 |  |  | 13.210 | |  |  |  |
| Group × Week × Minute | 9 | 1707.89 | * | 31.602 | 0.0049 |  |  | 19.893 |  |  | 63.297 |  |  | 18.272 | | * | 3.269 |  |
| Error | 155 | 767.90 |  |  | 0.0027 |  |  | 20.768 |  |  | 101.38 |  |  | 8.2805 | |  |  |  |

DF: Degree of freedom, MS: Mean Squares, LSD: Least significant difference. *: p<0.05, **: p<0.01, Glu: glucose, pH(T)-pCO_2_(T)-pO_2_(T): temperature corrected values according to 37 ˚C, p50: oxygen pressure in the semi-saturated blood.

**Supplemental Table S4.** The ANOVA table for the significance of treatments and their interactions according to factorial experiment design in randomized blocks for O_2_, Base(Ecf), Base(B), HCO_3_- (st) and HCO_3_-.

| **Sources** |  | **O_2_ (vol%)** | | | **Base(Ecf) (mmol/L)** | | | **Base(B) (mmol/L)** | | | | **HCO_3_- (st) (mmol/L)** | | | **HCO_3_- (mmol/L)** | | |
| --- | --- | --- | --- | --- | --- | --- | --- | --- | --- | --- | --- | --- | --- | --- | --- | --- | --- |
|  | DF | MS |  | LSD_0.05_ | MS |  | LSD_0.05_ | MS |  | LSD_0.05_ | MS | |  | LSD_0.05_ | MS |  | LSD_0.05_ |
| Block | 5 | 76.216 |  |  | 46.340 |  |  | 37.808 |  |  | 19.810 | |  |  | 40.600 |  |  |
| Group | 3 | 37.730 | * | 1.276 | 9.0956 | * | 0.703 | 11.617 | * | 0.774 | 7.6287 | | * | 0.691 | 10.269 | * | 0.756 |
| Week | 3 | 34.104 | * | 0.325 | 30.898 | ** | 0.703 | 34.391 | ** | 0.774 | 29.296 | | ** | 0.691 | 13.385 | * | 0.756 |
| Group × Week | 9 | 31.311 | ** | 2.552 | 11.367 | ** | 1.408 | 13.428 | ** | 1.550 | 13.702 | | ** | 1.382 | 10.171 | ** | 1.515 |
| Minute | 1 | 0.8086 |  |  | 0.7166 |  |  | 0.9520 |  |  | 0.2730 | |  |  | 0.3144 |  |  |
| Group × Minute | 3 | 0.8932 |  |  | 0.8279 |  |  | 3.3480 |  |  | 0.8407 | |  |  | 5.5012 |  |  |
| Week × Minute | 3 | 3.1830 |  |  | 8.3078 | * | 1.992 | 17.513 | ** | 1.096 | 3.1011 | |  |  | 7.1050 |  |  |
| Group × Week × Minute | 9 | 2.7148 |  |  | 6.7008 | * | 0.995 | 11.085 | ** | 2.194 | 5.2280 | |  |  | 3.5162 |  |  |
| Error | 155 | 10.030 |  |  | 3.0574 |  |  | 3.7055 |  |  | 2.9406 | |  |  | 3.5243 |  |  |

DF: Degree of freedom, MS: Mean Squares, LSD: Least significant difference. *: p<0.05, **: p<0.01, O_2_ (vol%): oxygen concentration, Base(Ecf): base status in extracellular fluid, Base(B): base status in blood, HCO_3_- (st): standard bicarbonate HCO_3_-: bicarbonate.

**Supplemental Table S5.** The ANOVA table for the significance of treatments and their interactions according to factorial experiment design in randomized blocks for K^+^, Na^+^ and Ca^+^.

| **Sources** |  | **K^+^ (mmol/L)** | | | **Na^+^ (mmol/L)** | | | **Ca^+^ (mmol/L)** | | |
| --- | --- | --- | --- | --- | --- | --- | --- | --- | --- | --- |
|  | DF | MS |  | LSD_0.05_ | MS |  | LSD_0.05_ | MS |  | LSD_0.05_ |
| Block | 5 | 0.9434 |  |  | 43.384 |  |  | 0.0184 |  |  |
| Group | 3 | 12.314 | ** | 0.140 | 24.389 | * | 1.204 | 0.1522 | ** | 0.023 |
| Week | 3 | 4.6595 | ** | 0.140 | 30.640 | * | 1.204 | 0.0289 | ** | 0.023 |
| Group × Week | 9 | 1.4227 | ** | 0.282 | 45.429 | ** | 2.541 | 0.0407 | ** | 0.047 |
| Minute | 1 | 0.3701 |  |  | 12.834 |  |  | 0.0009 |  |  |
| Group × Minute | 3 | 0.2118 |  |  | 6.9145 |  |  | 0.0100 | * | 0.034 |
| Week × Minute | 3 | 0.6887 | ** | 0.199 | 40.324 | ** | 1.704 | 0.0002 |  |  |
| Group × Week × Minute | 9 | 0.2409 |  |  | 16.203 |  |  | 0.0015 |  |  |
| Error | 155 | 0.1243 |  |  | 8.9562 |  |  | 0.0036 |  |  |

DF: Degree of freedom, MS: Mean Squares, LSD: Least significant difference. *: p<0.05, **: p<0.01, K^+^: potassium, Na^+^:sodium, Ca^+^: calcium.

**Supplemental Table S6**. Effects of experimental factors on investigated blood gas values

| **Groups** | **Weeks** | **pH** | | | **pCO_2_ (mmHg)** | | | **pO_2_ (mmHg)** | | |
| --- | --- | --- | --- | --- | --- | --- | --- | --- | --- | --- |
|  |  | **5 min** | **10 min** | **Mean** | **5 min** | **10 min** | **Mean** | **5 min** | **10 min** | **Mean** |
| C | I | 7.355^c-f^ | 7.365^b-e^ | 7.36^C^ | 45.97 | 45.62 | 45.79^B-E^ | 40.13 | 40.52 | 40.33^FG^ |
|  | II | 7.363^b-e^ | 7.372^b-e^ | 7.37^BC^ | 45.73 | 45.98 | 45.85^B-E^ | 40.52 | 40.58 | 40.55^EFG^ |
|  | III | 7.370^b-e^ | 7.372^b-e^ | 7.37^BC^ | 45.49 | 45.68 | 45.59^B-F^ | 40.37 | 40.30 | 40.33^FG^ |
|  | IV | 7.375^b-e^ | 7.373^b-e^ | 7.37^ABC^ | 45.96 | 46.04 | 46.00^B-E^ | 40.32 | 40.35 | 40.33^FG^ |
| CoQ_10_ | I | 7.355^c-f^ | 7.358^b-e^ | 7.36^C^ | 45.69 | 45.96 | 45.83^B-E^ | 40.40 | 40.62 | 40.51^EFG^ |
|  | II | 7.350^def^ | 7.372^b-e^ | 7.36^C^ | 49.02 | 48.52 | 48.77^ABC^ | 40.73 | 40.33 | 40.53^EFG^ |
|  | III | 7.347^def^ | 7.350^def^ | 7.35^C^ | 50.40 | 51.48 | 50.94^A^ | 46.22 | 46.45 | 46.33^DEF^ |
|  | IV | 7.348^def^ | 7.352^c-f^ | 7.35^C^ | 48.07 | 48.23 | 48.15^ABC^ | 59.89 | 60.04 | 59.96^A^ |
| HIIT | I | 7.273^h^ | 7.333^efg^ | 7.30^D^ | 44.06 | 44.06 | 44.06^DEF^ | 47.14 | 39.74 | 43.44^D-G^ |
|  | II | 7.393^abc^ | 7.350^def^ | 7.37^BC^ | 46.12 | 44.12 | 45.12^C-F^ | 43.08 | 49.85 | 46.47^DE^ |
|  | III | 7.355^c-f^ | 7.342^e-f^ | 7.35^C^ | 45.37 | 47.00 | 46.18^E^ | 42.67 | 51.40 | 47.03^CD^ |
|  | IV | 7.388^a-d^ | 7.352^c-f^ | 7.37^BC^ | 44.35 | 44.23 | 44.29^DEF^ | 45.27 | 42.22 | 43.74^D-G^ |
| HIIT+Q_10_ | I | 7.313^fgh^ | 7.292^gh^ | 7.30^D^ | 45.94 | 47.40 | 46.67^BCD^ | 60.20 | 45.70 | 52.95^BC^ |
|  | II | 7.398^ab^ | 7.360^b-e^ | 7.38^ABC^ | 39.80 | 43.88 | 41.84^F^ | 36.77 | 39.65 | 38.21^G^ |
|  | III | 7.370^b-e^ | 7.368^b-e^ | 7.37^BC^ | 47.77 | 50.88 | 49.33^AB^ | 42.46 | 48.52 | 45.49^DEF^ |
|  | IV | 7.388^a-d^ | 7.418^a^ | 7.40^A^ | 44.35 | 40.45 | 42.40^EF^ | 57.93 | 56.49 | 57.21^AB^ |
| Mean | | 7.359 | 7.361 | 7.36 | 45.88 | 46.22 | 46.05 | 45.25 | 45.17 | 45.21 |
|  | I | 7.324 | 7.337 | 7.33^C^ | 45.42 | 45.76 | 45.59^B^ | 46.97^ab^ | 41.64^d^ | 44.30^BC^ |
|  | II | 7.376 | 7.375 | 7.38^A^ | 45.17 | 45.62 | 45.39^B^ | 40.27^d^ | 42.60^cd^ | 41.43^C^ |
|  | III | 7.360 | 7.358 | 7.36^B^ | 47.26 | 48.76 | 48.01^A^ | 42.93^bcd^ | 46.67^abc^ | 44.79^B^ |
|  | IV | 7.375 | 7.374 | 7.37^AB^ | 45.68 | 44.74 | 45.21^B^ | 50.85^a^ | 49.7^a^ | 50.31^A^ |
| C |  | 7.366 | 7.370 | 7.37^A^ | 45.79 | 45.83 | 45.81^B^ | 40.33 | 40.44 | 40.39^C^ |
| CoQ_10_ |  | 7.350 | 7.358 | 7.35^AB^ | 48.29 | 48.55 | 48.42^A^ | 46.81 | 46.86 | 46.83^AB^ |
| HIIT |  | 7.353 | 7.356 | 7.34^B^ | 44.97 | 44.85 | 44.91^B^ | 44.54 | 45.80 | 45.17^B^ |
| HIIT+Q_10_ |  | 7.368 | 7.360 | 7.36^A^ | 44.46 | 45.65 | 45.06^B^ | 49.34 | 47.59 | 48.46^A^ |

C: Control group, CoQ_10_: Coenzyme Q_10_ supplementation group, HIIT: High intensity interval training group, HIIT+Q_10_: High intensity interval training group+coenzyme Q_10_ supplementation group, pCO_2_: partial pressure of carbon dioxide, pO_2_ partial pressure of oxygen. Levels not connected by same letter are significantly different.

**Supplemental Table S7**. Effects of experimental factors on investigated oximetry values

| **Groups** | **Weeks** | **Hb (g/dl)** | | | **HCT (%)** | | | **sO_2_ (%)** | | | **COHb (%)** | | | **HHb (%)** | | | | **MetHb (%)** | | |  |
| --- | --- | --- | --- | --- | --- | --- | --- | --- | --- | --- | --- | --- | --- | --- | --- | --- | --- | --- | --- | --- | --- |
|  |  | **5 min** | **10 min** | **Mean** | **5 min** | **10 min** | **Mean** | **5 min** | **10 min** | **Mean** | **5 min** | **10 min** | **Mean** | **5 min** | **10 min** | **Mean** | **5 min** | | **10 min** | **Mean** | |
| C | I | 14.03 | 14.08 | 14.06^EF^ | 41.95^hij^ | 41.95^hij^ | 41.95^EF^ | 50.92 | 48.42 | 49.67^D^ | 1.23 | 1.30 | 1.27^BC^ | 46.25 | 46.25 | 46.25^AB^ | 0.67 | | 0.62 | 0.64^BC^ | |
|  | II | 14.07 | 14.08 | 14.08^EF^ | 41.95^hij^ | 41.95^hij^ | 41.95^EF^ | 50.92 | 50.92 | 50.92^D^ | 1.37 | 1.57 | 1.47^B^ | 47.45 | 42.78 | 45.12^AB^ | 0.56 | | 0.58 | 0.57^BCD^ | |
|  | III | 14.07 | 14.08 | 14.08^EF^ | 41.85^hij^ | 41.88^hij^ | 41.87^EF^ | 49.35 | 50.92 | 50.13^D^ | 1.43 | 1.27 | 1.35^BC^ | 42.78 | 46.12 | 44.45^AB^ | 0.58 | | 0.57 | 0.58^BCD^ | |
|  | IV | 14.10 | 14.05 | 14.08^EF^ | 41.97^hij^ | 41.87^hij^ | 41.92^EF^ | 50.92 | 50.92 | 50.92^D^ | 1.23 | 1.22 | 1.23^BC^ | 43.57 | 44.32 | 43.94^AB^ | 0.58 | | 0.58 | 0.58^BCD^ | |
| CoQ_10_ | I | 14.03 | 14.08 | 14.06^EF^ | 41.95^hij^ | 41.95^hij^ | 41.95^EF^ | 50.92 | 50.92 | 50.92^D^ | 1.32 | 1.28 | 1.30^BC^ | 42.45 | 42.72 | 42.58^AB^ | 0.60 | | 0.59 | 0.60^BC^ | |
|  | II | 15.84 | 16.09 | 15.96^AB^ | 48.46^a-e^ | 48.52^a-d^ | 48.49^ABC^ | 62.33 | 65.67 | 64.00^ABC^ | 2.77 | 2.85 | 2.81^A^ | 35.64 | 35.64 | 35.64^BC^ | 0.37 | | 0.34 | 0.36^B-E^ | |
|  | III | 12.72 | 12.72 | 12.72^G^ | 40.54^hij^ | 42.39^g-j^ | 41.46^F^ | 52.42 | 52.58 | 52.50^CD^ | 1.35 | 1.20 | 1.28^BC^ | 51.63 | 52.47 | 52.05^A^ | 0.07 | | 0.11 | 0.09^EFG^ | |
|  | IV | 15.15 | 15.37 | 15.26^A-D^ | 45.50^c-ı^ | 45.17^c-ı^ | 45.33^B-E^ | 50.92 | 51.42 | 51.17^D^ | 1.47 | 1.47 | 1.47^B^ | 37.20 | 36.37 | 36.78^BC^ | 1.14 | | 1.20 | 1.17^A^ | |
| HIIT | I | 15.08 | 14.24 | 14.66^C-F^ | 40.90^hij^ | 41.70^hij^ | 41.30^F^ | 65.14 | 65.48 | 65.31^AB^ | 3.96 | 2.08 | 3.02^A^ | 30.54 | 44.68 | 37.61^BC^ | -0.02 | | -0.04 | -0.03^FG^ | |
|  | II | 16.77 | 14.77 | 15.77^ABC^ | 51.32^a^ | 44.80^d-i^ | 48.06^ABC^ | 47.30 | 65.37 | 56.33^BCD^ | 1.22 | 1.97 | 1.59^B^ | 52.35 | 48.15 | 50.25^A^ | 0.13 | | 0.27 | 0.20^D-G^ | |
|  | III | 14.04 | 15.48 | 14.76^C-F^ | 43.04^f-j^ | 47.35^a-g^ | 45.20^CDE^ | 51.33 | 61.98 | 56.66^BCD^ | 0.70 | 1.45 | 1.08^BC^ | 48.52 | 37.35 | 42.93^A^ | -0.37 | | 0.60 | 0.12^EFG^ | |
|  | IV | 14.17 | 14.76 | 14.46^DEF^ | 43.50^e-j^ | 45.22^c-i^ | 44.36^DEF^ | 56.77 | 52.42 | 54.59^BCD^ | 1.45 | 0.56 | 1.01^BC^ | 42.55 | 47.30 | 44.93^AB^ | 0.57 | | 0.16 | 0.36^B-E^ | |
| HIIT+Q_10_ | I | 14.46 | 12.76 | 13.61^FG^ | 45.86^b-h^ | 39.22^j^ | 42.54^EF^ | 58.26 | 52.84 | 55.55^BCD^ | 1.66 | 1.12 | 1.39^BC^ | 40.08 | 46.44 | 43.26^AB^ | 0.72 | | 0.70 | 0.71^B^ | |
|  | II | 14.55 | 15.43 | 14.99^B-E^ | 44.57^d-i^ | 50.13^abc^ | 47.35^A-D^ | 47.38 | 46.72 | 47.05^D^ | 0.70 | 0.75 | 0.73^BC^ | 52.33 | 54.63 | 53.48^A^ | -0.05 | | -0.20 | -0.13^G^ | |
|  | III | 16.17 | 16.55 | 16.36^A^ | 49.55^a-d^ | 50.70^ab^ | 50.13^A^ | 53.73 | 48.70 | 51.22^D^ | 0.32 | 0.75 | 0.53^C^ | 46.40 | 54.15 | 50.28^A^ | -0.22 | | 0.78 | 0.28^C-F^ | |
|  | IV | 15.53 | 16.34 | 15.93^AB^ | 47.59^a-f^ | 49.98^abc^ | 48.78^AB^ | 78.18 | 72.63 | 75.41^A^ | 1.48 | 1.78 | 1.63^B^ | 26.10 | 31.64 | 28.87^C^ | 0.61 | | 0.72 | 0.67^BC^ | |
| Mean | | 14.67 | 14.68 | 14.68 | 44.41 | 44.67 | 44.54 | 54.80 | 55.49 | 55.15 | 1.48 | 1.41 | 1.45 | 42.87 | 44.44 | 43.65 | 0.37 | | 0.47 | 0.42 | |
|  | I | 14.40 | 13.79 | 14.10^C^ | 42.66 | 41.21 | 41.93^C^ | 56.31 | 54.41 | 55.36 | 2.04 | 1.45 | 1.74^A^ | 39.83 | 45.02 | 42.42^AB^ | 0.49^ab^ | | 0.47^ab^ | 0.48^B^ | |
|  | II | 15.31 | 15.09 | 15.20^A^ | 46.57 | 46.35 | 46.46^A^ | 51.98 | 57.17 | 54.58 | 1.51 | 1.78 | 1.65^A^ | 46.94 | 45.30 | 46.12^A^ | 0.26^bc^ | | 0.25^bc^ | 0.25^C^ | |
|  | III | 14.25 | 14.71 | 14.48^BC^ | 43.75 | 45.58 | 44.66^B^ | 51.71 | 53.54 | 52.63 | 0.95 | 1.17 | 1.06^B^ | 47.33 | 47.52 | 47.42^A^ | 0.02^c^ | | 0.52^ab^ | 0.27^C^ | |
|  | IV | 14.74 | 15.13 | 14.93^AB^ | 44.64 | 45.56 | 45.09^AB^ | 59.19 | 56.85 | 58.02 | 1.41 | 1.26 | 1.33^AB^ | 37.35 | 39.91 | 38.62^B^ | 0.72^a^ | | 0.67^a^ | 0.69^A^ | |
| C |  | 14.07 | 14.08 | 14.07^C^ | 41.93 | 41.91 | 41.92^C^ | 50.53 | 50.29 | 50.41^B^ | 1.32 | 1.34 | 1.33^AB^ | 45.01 | 44.87 | 44.94 | 0.60 | | 0.59 | 0.59^A^ | |
| CoQ_10_ |  | 14.44 | 14.57 | 14.50^BC^ | 44.11 | 44.51 | 44.30^B^ | 54.15 | 55.15 | 54.65^AB^ | 1.73 | 1.70 | 1.71^A^ | 41.73 | 41.80 | 41.76 | 0.55 | | 0.56 | 0.55^AB^ | |
| HIIT |  | 15.01 | 14.81 | 14.91^AB^ | 44.69 | 44.77 | 44.72^B^ | 55.14 | 61.31 | 58.22^A^ | 1.83 | 1.51 | 1.67^A^ | 43.49 | 44.37 | 43.93 | 0.08 | | 0.25 | 0.16^C^ | |
| HIIT+Q_10_ |  | 15.18 | 15.27 | 15.22^A^ | 46.89 | 47.51 | 47.19^A^ | 59.39 | 55.22 | 57.31^A^ | 1.04 | 1.10 | 1.07^B^ | 41.23 | 46.72 | 43.97 | 0.27 | | 0.50 | 0.38^B^ | |

C: Control group, CoQ_10_: Coenzyme Q_10_ supplementation group, HIIT: High intensity interval training group, HIIT+Q_10_: High intensity interval training group+coenzyme Q_10_ supplementation group, Hb: hemoglobin, HCT: hematokrit, sO_2_: oxygen saturation, COHb: [carboxyhemoglobin](https://tureng.com/tr/turkce-ingilizce/carboxyhemoglobin), HHb: deoxyhemoglobin, MetHb: methemoglobin. Levels not connected by same letter are significantly different.

**Supplemental Table S8**. Effects of experimental factors on investigated metabolite values

| **Groups** | **Weeks** | **Lac (mmol/L)** | | | **Glu (mg/dl)** | | |
| --- | --- | --- | --- | --- | --- | --- | --- |
|  |  | **5 min** | **10 min** | **Mean** | **5 min** | **10 min** | **Mean** |
| C | I | 3.54^f^ | 3.51^f^ | 3.52^DE^ | 170.23^fgh^ | 169.00^fgh^ | 169.62^E^ |
|  | II | 3.57^f^ | 3.54^f^ | 3.56^D^ | 169.80^fgh^ | 169.67^fgh^ | 169.73^E^ |
|  | III | 3.54^f^ | 3.56^f^ | 3.55^D^ | 169.83^fgh^ | 170.33^fgh^ | 170.08^E^ |
|  | IV | 3.54^f^ | 3.56^f^ | 3.55^D^ | 170.50^fgh^ | 170.73^fgh^ | 170.62^E^ |
| CoQ_10_ | I | 3.56^f^ | 3.54^f^ | 3.55^D^ | 169.00^fgh^ | 169.80^fgh^ | 169.40^E^ |
|  | II | 2.02^hij^ | 2.02^hij^ | 2.02^HI^ | 107.33^j^ | 107.33^j^ | 107.33^G^ |
|  | III | 2.58^gh^ | 2.56^gh^ | 2.57^FG^ | 214.36^cd^ | 214.36^cd^ | 214.36^D^ |
|  | IV | 3.67^ef^ | 3.66^ef^ | 3.66^D^ | 258.70^a^ | 260.59^a^ | 259.65^A^ |
| HIIT | I | 7.22^b^ | 4.50^d^ | 5.86^A^ | 168.00^fgh^ | 194.00^def^ | 181.00^E^ |
|  | II | 5.85^c^ | 3.07^fg^ | 4.46^C^ | 143.00^hi^ | 122.00^ij^ | 132.50^F^ |
|  | III | 7.96^a^ | 3.63^ef^ | 5.79^A^ | 224.94^bcd^ | 249.50^ab^ | 237.22^BC^ |
|  | IV | 5.75^c^ | 4.68^d^ | 5.22^B^ | 168.83^fgh^ | 182.20^efg^ | 175.52^E^ |
| HIIT+Q_10_ | I | 6.06^c^ | 4.36^de^ | 5.21^B^ | 146.60^hi^ | 214.40^cd^ | 180.50^E^ |
|  | II | 1.37^j^ | 1.70^ij^ | 1.53^I^ | 160.50^gh^ | 193.83^def^ | 177.17^E^ |
|  | III | 2.42^ghi^ | 2.52^gh^ | 2.47^GH^ | 258.00^a^ | 239.83^abc^ | 248.92^AB^ |
|  | IV | 2.35^ghi^ | 3.66^ef^ | 3.01^EF^ | 223.78^bcd^ | 208.70^cde^ | 216.24^CD^ |
| Mean | | 4.06^A^ | 3.38^B^ | 3.72 | 182.71 | 189.77 | 186.24 |
|  | I | 5.09^a^ | 3.98^b^ | 4.54^A^ | 163.46 | 186.80 | 175.13^C^ |
|  | II | 3.20^c^ | 2.58^d^ | 2.89^D^ | 145.16 | 148.21 | 146.68^D^ |
|  | III | 4.12^b^ | 3.07^c^ | 3.59^C^ | 216.78 | 218.51 | 217.64^A^ |
|  | IV | 3.83^b^ | 3.89^b^ | 3.86^B^ | 205.45 | 205.56 | 205.50^B^ |
| C |  | 3.55^c^ | 3.54^c^ | 3.55^B^ | 170.09 | 169.93 | 170.01^C^ |
| CoQ_10_ |  | 2.96^d^ | 2.94^d^ | 2.95^C^ | 187.35 | 188.02 | 187.68^B^ |
| HIIT |  | 6.70^a^ | 3.97^b^ | 5.33^A^ | 176.19 | 186.93 | 181.56^B^ |
| HIIT+Q_10_ |  | 3.05^d^ | 3.06^d^ | 3.05^C^ | 197.22 | 214.19 | 205.71^A^ |

C: Control group, CoQ_10_: Coenzyme Q_10_ supplementation group, HIIT: High intensity interval training group, HIIT+Q_10_: High intensity interval training group+coenzyme Q_10_ supplementation group, Lac: lactate. Glu: glucose. Levels not connected by same letter are significantly different.

**Supplemental Table S9**. Effects of experimental factors on investigated temperature corrected values

| **Groups** | **Weeks** | **pH(T)** | | | **pCO_2_(T) (mmHg)** | | | **pO_2_(T) (mmHg)** | | |
| --- | --- | --- | --- | --- | --- | --- | --- | --- | --- | --- |
|  |  | **5 min** | **10 min** | **Mean** | **5 min** | **10 min** | **Mean** | **5 min** | **10 min** | **Mean** |
| C | I | 7.38 | 7.38 | 7.38^ABC^ | 45.28 | 45.16 | 45.22^BCD^ | 39.82 | 39.52 | 39.67^CD^ |
|  | II | 7.38 | 7.38 | 7.38^ABC^ | 45.21 | 44.99 | 45.10^CD^ | 39.82 | 39.69 | 39.75^CD^ |
|  | III | 7.38 | 7.38 | 7.38^ABC^ | 45.01 | 45.01 | 45.01^CD^ | 39.52 | 39.69 | 39.60^CD^ |
|  | IV | 7.38 | 7.38 | 7.38^ABC^ | 45.01 | 45.01 | 45.01^CD^ | 39.52 | 39.52 | 39.52^CD^ |
| CoQ_10_ | I | 7.38 | 7.38 | 7.38^ABC^ | 45.33 | 45.24 | 45.29^BCD^ | 39.69 | 39.69 | 39.69^CD^ |
|  | II | 7.35 | 7.35 | 7.35^BC^ | 48.45 | 48.38 | 48.42^ABC^ | 47.41 | 47.16 | 47.28^BC^ |
|  | III | 7.34 | 7.34 | 7.34^CD^ | 48.27 | 48.82 | 48.54^ABC^ | 46.22 | 46.05 | 46.13^BCD^ |
|  | IV | 7.34 | 7.35 | 7.34^CD^ | 48.30 | 48.37 | 48.33^ABC^ | 58.42 | 58.41 | 58.41^A^ |
| HIIT | I | 7.20 | 7.32 | 7.26^E^ | 52.78 | 44.86 | 48.82^AB^ | 51.58 | 54.40 | 52.98^AB^ |
|  | II | 7.38 | 7.40 | 7.39^AB^ | 46.12 | 44.12 | 45.12^CD^ | 43.08 | 49.85 | 46.46^BC^ |
|  | III | 7.35 | 7.34 | 7.34^CD^ | 45.37 | 47.00 | 46.18^ABC^ | 42.67 | 48.11 | 45.38^BCD^ |
|  | IV | 7.39 | 7.36 | 7.37^ABC^ | 44.35 | 46.68 | 45.52^BCD^ | 57.93 | 61.59 | 59.75^A^ |
| HIIT+Q_10_ | I | 7.31 | 7.29 | 7.30^D^ | 45.94 | 47.40 | 46.67^ABC^ | 60.20 | 45.70 | 52.95^AB^ |
|  | II | 7.40 | 7.39 | 7.39^A^ | 39.80 | 43.88 | 41.84^DE^ | 36.77 | 39.65 | 38.20^D^ |
|  | III | 7.32 | 7.37 | 7.34^CD^ | 47.77 | 50.88 | 49.33^A^ | 42.46 | 48.53 | 45.49^BCD^ |
|  | IV | 7.39 | 7.42 | 7.40^A^ | 41.08 | 40.45 | 40.76^E^ | 57.93 | 61.59 | 59.75^A^ |
| Mean | | 7.35 | 7.36 | 7.36 | 45.88 | 46.02 | 45.95 | 46.44 | 47.45 | 46.94 |
|  | I | 7.32 | 7.34 | 7.33^C^ | 47.33 | 45.67 | 46.49^AB^ | 47.82 | 44.83 | 46.32^B^ |
|  | II | 7.38 | 7.38 | 7.38^A^ | 44.89 | 45.34 | 45.11^B^ | 41.77 | 44.09 | 42.92^B^ |
|  | III | 7.35 | 7.36 | 7.35^B^ | 46.60 | 47.93 | 47.26^A^ | 42.72 | 45.59 | 44.15^B^ |
|  | IV | 7.38 | 7.38 | 7.38^A^ | 44.69 | 45.13 | 44.90^B^ | 53.45 | 55.28 | 54.36^A^ |
| C |  | 7.38 | 7.38 | 7.38^A^ | 45.13 | 45.04 | 45.08^B^ | 39.67 | 39.61 | 39.64^B^ |
| CoQ_10_ |  | 7.35 | 7.36 | 7.35^B^ | 47.59 | 47.70 | 47.64^A^ | 47.93 | 47.83 | 47.88^A^ |
| HIIT |  | 7.33 | 7.35 | 7.34^B^ | 47.15 | 45.66 | 46.40^AB^ | 48.81 | 53.49 | 51.15^A^ |
| HIIT+Q_10_ |  | 7.35 | 7.37 | 7.36^AB^ | 43.65 | 45.65 | 44.64^B^ | 49.34 | 48.87 | 49.10^A^ |

C: Control group, CoQ_10_: Coenzyme Q_10_ supplementation group, HIIT: High intensity interval training group, HIIT+Q_10_: High intensity interval training group+coenzyme Q_10_ supplementation group, pH(T)-pCO_2_(T)-pO_2_(T): temperature corrected values according to 37 ˚C. Levels not connected by same letter are significantly different.

**Supplemental Table S10**. Effects of experimental factors on investigated oxygen status values

| **Groups** | **Weeks** | **p50 (mmHg)** | | | **O_2_ (vol%)** | | |
| --- | --- | --- | --- | --- | --- | --- | --- |
|  |  | **5 min** | **10 min** | **Mean** | **5 min** | **10 min** | **Mean** |
| C | I | 34.85^gh^ | 35.17^e-h^ | 35.01^F^ | 11.35 | 11.17 | 11.26^DEF^ |
|  | II | 35.57^d-h^ | 35.23^e-h^ | 35.40^EF^ | 11.25 | 11.17 | 11.21^DEF^ |
|  | III | 35.57^d-h^ | 35.24^e-h^ | 35.40^EF^ | 11.23 | 10.77 | 11.00^DEF^ |
|  | IV | 34.93^fgh^ | 35.59^d-h^ | 35.26^EF^ | 10.98 | 10.93 | 10.96^DEF^ |
| CoQ_10_ | I | 35.06^e-h^ | 35.72^d-h^ | 35.39^EF^ | 10.82 | 10.80 | 10.81^EF^ |
|  | II | 38.01^c-g^ | 38.02^c-g^ | 38.01^BCD^ | 12.80 | 13.40 | 13.10^B-E^ |
|  | III | 41.69^ab^ | 41.71^ab^ | 41.70^A^ | 13.52 | 13.50 | 13.51^BCD^ |
|  | IV | 38.18^c-f^ | 38.22^cde^ | 38.20^BCD^ | 15.07 | 14.88 | 14.98^AB^ |
| HIIT | I | 38.15^c-f^ | 34.34^h^ | 36.25^DEF^ | 13.70 | 14.36 | 14.03^ABC^ |
|  | II | 37.90^c-g^ | 36.83^c-h^ | 37.37^CDE^ | 12.57 | 12.73 | 12.65^B-F^ |
|  | III | 41.60^ab^ | 36.83^c-h^ | 39.22^BC^ | 11.43 | 12.73 | 12.08^C-F^ |
|  | IV | 39.77^bc^ | 34.78^gh^ | 37.27^C-F^ | 12.45 | 12.20 | 12.33^C-F^ |
| HIIT+Q_10_ | I | 43.59^a^ | 35.78^d-h^ | 39.68^AB^ | 13.70 | 11.54 | 12.62^B-F^ |
|  | II | 38.05^c-g^ | 38.68^bcd^ | 38.37^BCD^ | 10.08 | 10.88 | 10.48^F^ |
|  | III | 38.05^c-g^ | 39.90^bc^ | 38.98^BC^ | 10.08 | 12.16 | 11.12^DEF^ |
|  | IV | 36.21^d-h^ | 36.07^d-h^ | 36.14^DEF^ | 16.41 | 16.29 | 16.35^A^ |
| Mean | | 37.95^A^ | 36.75^B^ | 37.35 | 12.34 | 12.47 | 12.40 |
|  | I | 37.91 | 35.25 | 36.58^B^ | 12.39 | 11.97 | 12.18^B^ |
|  | II | 37.38 | 37.19 | 37.29^B^ | 11.68 | 12.05 | 11.86^B^ |
|  | III | 39.26 | 38.42 | 38.82^A^ | 11.57 | 12.29 | 11.93^B^ |
|  | IV | 37.27 | 36.16 | 36.72^B^ | 13.73 | 13.58 | 13.65^A^ |
| C |  | 35.23^c^ | 35.31^c^ | 35.27^B^ | 11.20 | 11.01 | 11.11^B^ |
| CoQ_10_ |  | 38.24^ab^ | 38.42^ab^ | 38.33^A^ | 13.05 | 13.15 | 13.10^A^ |
| HIIT |  | 39.35^a^ | 35.70^c^ | 37.52^A^ | 12.54 | 13.01 | 12.77^A^ |
| HIIT+Q_10_ |  | 38.97^ab^ | 37.61^b^ | 38.29^A^ | 12.57 | 12.72 | 12.64^A^ |

C: Control group, CoQ_10_: Coenzyme Q_10_ supplementation group, HIIT: High intensity interval training group, HIIT+Q_10_: High intensity interval training group+coenzyme Q_10_ supplementation group, O_2_ (vol%): oxygen concentration, p50: oxygen pressure in the semi-saturated blood. Levels not connected by same letter are significantly different.

**Supplemental Table S11**. Effects of experimental factors on investigated acid-base status values

| **Groups** | **Weeks** | **Base(Ecf) (mmol/L)** | | | **Base(B) (mmol/L)** | | | **HCO_3_- (st) (mmol/L)** | | | **HCO_3_- (mmol/L)** | | |
| --- | --- | --- | --- | --- | --- | --- | --- | --- | --- | --- | --- | --- | --- |
|  |  | **5 min** | **10 min** | **Mean** | **5 min** | **10 min** | **Mean** | **5 min** | **10 min** | **Mean** | **5 min** | **10 min** | **Mean** |
| C | I | 0.69^c-f^ | 0.43^c-g^ | 0.56^CA^ | 0.43^b-g^ | 0.63^a-g^ | 0.53^AB^ | 23.87 | 23.30 | 23.58^ABC^ | 25.90 | 26.10 | 26.00^A-D^ |
|  | II | 0.69^c-f^ | 1.19 ^b-e^ | 0.94^ABC^ | 0.43^b-g^ | 0.77^a-g^ | 0.60^AB^ | 23.90 | 23.83 | 23.86^ABC^ | 26.07 | 25.48 | 25.77^A-D^ |
|  | III | 1.36^bcd^ | 0.63^c-f^ | 0.99^AB^ | 0.62^a-g^ | 0.50^a-g^ | 0.56^AB^ | 23.70 | 23.43 | 23.56^ABC^ | 25.97 | 25.52 | 25.74^A-D^ |
|  | IV | 0.69^c-f^ | 0.73^c-f^ | 0.71^BC^ | 0.43^b-g^ | 0.33^b-g^ | 0.38^ABC^ | 23.72 | 23.65 | 23.68^ABC^ | 26.12 | 25.85 | 25.98^A-D^ |
| CoQ_10_ | I | 0.88^b-f^ | 0.58^c-f^ | 0.73^BC^ | 0.25^c-g^ | 0.45^a-g^ | 0.35^ABC^ | 23.95 | 23.98 | 23.96^AB^ | 25.97 | 25.55 | 25.75^A-D^ |
|  | II | 0.95^b-f^ | 1.50^bcd^ | 1.23^AB^ | 0.88^a-f^ | 0.87^a-f^ | 0.88^AB^ | 24.30 | 24.35 | 24.32^A^ | 25.78 | 25.88 | 25.83^A-D^ |
|  | III | 1.13^b-f^ | 1.25^b-e^ | 1.19^AB^ | 0.28^b-g^ | 0.50^a-g^ | 0.39^AB^ | 22.88 | 22.87 | 22.87^BC^ | 25.77 | 25.70 | 25.73^A-D^ |
|  | IV | 1.75^abc^ | 1.72^a-d^ | 1.73^AB^ | 0.72^a-g^ | 0.72^a-g^ | 0.72^AB^ | 23.73 | 23.75 | 23.74^ABC^ | 26.50 | 26.50 | 26.50^AB^ |
| HIIT | I | -0.85^fg^ | -1.44^g^ | -1.15^DE^ | 0.04^c-g^ | -4.58^h^ | -2.27^DE^ | 21.80 | 21.10 | 21.45^DE^ | 24.76 | 21.94 | 23.35^F^ |
|  | II | 1.58^a-d^ | 2.87^ab^ | 2.23^A^ | 0.93^a-e^ | 2.63^a^ | 1.78^A^ | 23.93 | 25.82 | 24.87^A^ | 26.28 | 26.40 | 26.34^ABC^ |
|  | III | -0.68^efg^ | -0.25^d-g^ | -0.47^CD^ | -1.36^g^ | -0.98^efg^ | -1.17^CD^ | 22.39 | 22.75 | 22.57^CD^ | 24.55 | 24.75 | 24.65^DEF^ |
|  | IV | 1.70^a-d^ | 0.68^c-f^ | 1.19^AB^ | 1.30^a-d^ | 0.12^c-g^ | 0.71^AB^ | 24.58 | 23.54 | 24.06^AB^ | 26.18 | 25.54 | 25.86^A-D^ |
| HIIT+Q_10_ | I | -0.64^efg^ | -3.72^h^ | -2.18^E^ | -1.27^fg^ | -4.42^h^ | -2.84^E^ | 20.80 | 19.98 | 20.39^E^ | 24.58 | 23.03 | 23.80^EF^ |
|  | II | 0.67^cf^ | 0.47^c-g^ | 0.57^BC^ | 0.85^a-f^ | -0.67^d-g^ | 0.09^BC^ | 23.83 | 22.00 | 22.91^BC^ | 24.93 | 25.32 | 25.12^B-E^ |
|  | III | 0.02^c-g^ | 3.53^a^ | 1.78^AB^ | -1.12^efg^ | 2.45^ab^ | 0.67^AB^ | 22.38 | 25.22 | 23.80^ABC^ | 25.43 | 28.48 | 26.95^A^ |
|  | IV | -0.16^c-g^ | 1.60^a-d^ | 0.72^BC^ | -0.26^c-g^ | 1.59^abc^ | 0.66^AB^ | 23.71 | 25.12 | 24.41^A^ | 24.16 | 25.62 | 24.89^CDE^ |
| Mean | | 0.61 | 0.73 | 0.67 | 0.20 | 0.06 | 0.13 | 23.34 | 23.42 | 23.38 | 25.47 | 25.29 | 25.51 |
|  | I | 0.02^c^ | -1.04^d^ | -0.50^B^ | -0.14^ab^ | -1.98^c^ | -1.06^B^ | 22.60 | 22.09 | 22.34^C^ | 25.30 | 24.15 | 24.72^B^ |
|  | II | 0.97^abc^ | 1.51^a^ | 1.24^A^ | 0.78^a^ | 0.90^a^ | 0.83^A^ | 23.99 | 24.00 | 23.99^A^ | 25.77 | 25.77 | 25.76^A^ |
|  | III | 0.46^bc^ | 1.29^ab^ | 0.87^A^ | -0.39^b^ | 0.62^ab^ | 0.11^A^ | 22.84 | 23.57 | 23.20^B^ | 25.43 | 26.11 | 25.77^A^ |
|  | IV | 1.00^abc^ | 1.18^ab^ | 1.08^A^ | 0.55^ab^ | 0.69^ab^ | 0.62^A^ | 23.94 | 24.02 | 23.97^A^ | 25.74 | 25.88 | 25.80^A^ |
| C |  | 0.86 | 0.74 | 0.80^AB^ | 0.48 | 0.56 | 0.52^A^ | 23.80 | 23.55 | 23.67^A^ | 26.01 | 25.74 | 25.87^AB^ |
| CoQ_10_ |  | 1.18 | 1.26 | 1.21^A^ | 0.53 | 0.63 | 0.58^A^ | 23.72 | 23.74 | 23.72^A^ | 26.00 | 25.91 | 25.95^A^ |
| HIIT |  | 0.44 | 0.46 | 0.45^B^ | 0.23 | -0.70 | -0.24^B^ | 23.18 | 23.30 | 23.24^AB^ | 25.45 | 24.66 | 25.05^C^ |
| HIIT+Q_10_ |  | -0.03 | 0.47 | 0.22^B^ | -0.45 | -0.26 | -0.36^B^ | 22.68 | 23.08 | 22.88^B^ | 24.78 | 25.61 | 25.19^BC^ |

C: Control group, CoQ_10_: Coenzyme Q_10_ supplementation group, HIIT: High intensity interval training group, HIIT+Q_10_: High intensity interval training group+coenzyme Q_10_ supplementation group, Base(Ecf): base status in extracellular fluid, Base(B): base status in blood, HCO_3_- (st): standard bicarbonate HCO_3_-: bicarbonate. Levels not connected by same letter are significantly different.

**Supplemental Table S12**. Effects of experimental factors on investigated electrolyte values

| **Groups** | **Weeks** | **K^+^ (mmol/L)** | | | **Na^+^ (mmol/L)** | | | **Ca^+^ (mmol/L)** | | |
| --- | --- | --- | --- | --- | --- | --- | --- | --- | --- | --- |
|  |  | **5 min** | **10 min** | **Mean** | **5 min** | **10 min** | **Mean** | **5 min** | **10 min** | **Mean** |
| C | I | 4.65 | 4.58 | 4.62^A^ | 141.83 | 142.00 | 141.92^C-F^ | 1.43 | 1.45 | 1.44^AB^ |
|  | II | 4.63 | 4.67 | 4.65^A^ | 142.17 | 142.33 | 142.25^C-F^ | 1.47 | 1.49 | 1.48^A^ |
|  | III | 4.70 | 4.73 | 4.72^A^ | 142.50 | 142.67 | 142.58^B-E^ | 1.41 | 1.41 | 1.41^BC^ |
|  | IV | 4.55 | 4.62 | 4.58^A^ | 142.83 | 143.00 | 142.92^BCD^ | 1.47 | 1.49 | 1.48^A^ |
| CoQ_10_ | I | 4.65 | 4.68 | 4.67^A^ | 143.17 | 143.33 | 143.25^BC^ | 1.45 | 1.49 | 1.47^A^ |
|  | II | 4.65 | 4.58 | 4.62^A^ | 142.83 | 142.83 | 142.83^BCD^ | 1.46 | 1.47 | 1.46^A^ |
|  | III | 3.22 | 3.22 | 3.22^E^ | 143.67 | 144.00 | 143.83^BC^ | 1.35 | 1.37 | 1.36^DEF^ |
|  | IV | 3.73 | 3.75 | 3.74^C^ | 141.67 | 141.33 | 141.50^C-F^ | 1.35 | 1.35 | 1.35^EF^ |
| HIIT | I | 4.46 | 3.64 | 4.05^B^ | 139.00 | 141.00 | 140.00^F^ | 1.41 | 1.34 | 1.38^CDE^ |
|  | II | 3.25 | 3.38 | 3.32^DE^ | 144.33 | 136.17 | 140.25^EF^ | 1.35 | 1.29 | 1.32^FG^ |
|  | III | 3.31 | 3.23 | 3.27^DE^ | 140.09 | 141.25 | 140.67^DEF^ | 1.39 | 1.38 | 1.38^CDE^ |
|  | IV | 3.67 | 3.34 | 3.50^CD^ | 145.33 | 144.60 | 144.97^AB^ | 1.39 | 1.35 | 1.37^C-F^ |
| HIIT+Q_10_ | I | 4.48 | 3.66 | 4.07^B^ | 145.20 | 148.00 | 146.60^A^ | 1.41 | 1.40 | 1.41^BCD^ |
|  | II | 3.45 | 3.95 | 3.70^C^ | 142.17 | 138.00 | 140.08^F^ | 1.23 | 1.22 | 1.22^H^ |
|  | III | 3.38 | 3.40 | 3.39^DE^ | 143.00 | 142.00 | 142.50^CDE^ | 1.38 | 1.35 | 1.36^C-F^ |
|  | IV | 3.35 | 3.30 | 3.33^DE^ | 143.66 | 142.66 | 143.16^BC^ | 1.29 | 1.31 | 1.30^G^ |
| Mean | | 4.01 | 3.92 | 3.96 | 142.72 | 142.20 | 142.46 | 1.39 | 1.39 | 1.39 |
|  | I | 4.56^a^ | 4.14^b^ | 4.35^A^ | 142.30^a^ | 143.58^a^ | 142.94^A^ | 1.43 | 1.42 | 1.42^A^ |
|  | II | 4.00^bc^ | 4.15^b^ | 4.07^B^ | 142.88^a^ | 139.83^b^ | 141.35^B^ | 1.38 | 1.37 | 1.37^B^ |
|  | III | 3.65^d^ | 3.64^d^ | 3.65^C^ | 142.32^a^ | 142.48^a^ | 142.40^AB^ | 1.38 | 1.38 | 1.38^B^ |
|  | IV | 3.83^cd^ | 3.75^d^ | 3.79^C^ | 143.37^a^ | 142.90^a^ | 143.14^A^ | 1.37 | 1.37 | 1.37^B^ |
| C |  | 4.63 | 4.65 | 4.64^A^ | 142.33 | 142.50 | 142.42^AB^ | 1.45^ab^ | 1.46^a^ | 1.45^A^ |
| CoQ_10_ |  | 4.06 | 4.06 | 4.06^B^ | 142.83 | 142.88 | 142.85^A^ | 1.40^c^ | 1.42^bc^ | 1.41^B^ |
| HIIT |  | 3.67 | 3.40 | 3.54^C^ | 142.19 | 140.75 | 141.47^B^ | 1.39^c^ | 1.34^d^ | 1.36^C^ |
| HIIT+Q_10_ |  | 3.67 | 3.58 | 3.62^C^ | 143.51 | 142.67 | 143.09^A^ | 1.33^d^ | 1.32^d^ | 1.32^D^ |

C: Control group, CoQ_10_: Coenzyme Q_10_ supplementation group, HIIT: High intensity interval training group, HIIT+Q_10_: High intensity interval training group+coenzyme Q_10_ supplementation group, K^+^: potassium, Na^+^: sodium, Ca^+^: calcium. Levels not connected by same letter are significantly different.
